# Organotypic stromal cells impact endothelial cell transcriptome in 3D microvessel networks

**DOI:** 10.1038/s41598-022-24013-y

**Published:** 2022-11-28

**Authors:** Matthew B. Curtis, Natalie Kelly, Christopher C. W. Hughes, Steven C. George

**Affiliations:** 1 Department of Biomedical Engineering, University of California, Davis, 451 E. Health Sciences Drive, Room 2315, Davis, CA 95616 USA; 2 Department of Biomedical Engineering, University of California Irvine, Irvine, CA USA; 3 Department of Molecular Biology and Biochemistry, University of California Irvine, Irvine, CA USA

**Keywords:** Tissue engineering, Computational biology and bioinformatics

## Abstract

Endothelial cells line all major blood vessels and serve as integral regulators of many functions including vessel diameter, cellular trafficking, and transport of soluble mediators. Despite similar functions, the phenotype of endothelial cells is highly organ-specific, yet our understanding of the mechanisms leading to organ-level differentiation is incomplete. We generated 3D microvessel networks by combining a common naïve endothelial cell with six different stromal cells derived from the lung, skin, heart, bone marrow, pancreas, and pancreatic cancer. Single cell RNA-Seq analysis of the microvessel networks reveals five distinct endothelial cell populations, for which the relative proportion depends on the stromal cell population. Morphologic features of the organotypic vessel networks inversely correlate with a cluster of endothelial cells associated with protein synthesis. The organotypic stromal cells were each characterized by a unique subpopulation of cells dedicated to extracellular matrix organization and assembly. Finally, compared to cells in 2D monolayer, the endothelial cell transcriptome from the 3D in vitro heart, skin, lung, and pancreas microvessel networks are more similar to the in vivo endothelial cells from the respective organs. We conclude that stromal cells contribute to endothelial cell and microvessel network organ tropism, and create an endothelial cell phenotype that more closely resembles that present in vivo.

## Introduction

Endothelial cells (ECs) line the vasculature and directly impact the structure and function of all vessels, including capillaries. Interestingly, the structure and function of capillary beds is markedly organ-specific, and generally contributes to the overall function of the organ^1^. For example, the capillary endothelium in the central nervous system is characterized by a reduced ability of molecules to transit from the blood to the tissue, and vice versa (i.e., the “blood–brain barrier”)^2^. In contrast, the capillary endothelium in the liver exhibits large fenestrations which allow molecules to easily pass between the tissue and the blood^3^. In addition, increasing evidence shows that the vasculature also plays a role in patterning tissues^4–6^, suggesting bi-directional crosstalk between the endothelium and the surrounding parenchyma. Much work has been done to characterize organ-specific ECs, but the mechanism for how these organ-specific differences emerge is unclear^7–10^.

The local tissue microenvironment, including organotypic stromal cells (OSC), can potentially contribute to organ-specific capillary network structure and function. Stromal cells are a heterogeneous population of cells that form an integral part of the tissue microenvironment. Stromal cells perform diverse functions in normal physiology and pathology, including extracellular matrix (ECM) production and remodeling, and secretion of growth factors and cytokines, both of which can potentially impact EC phenotype^11,12^. Some stromal cells, such as pericytes, directly associate with blood vessels, and can regulate capillary permeability and sprouting angiogenesis^13–17^. Additionally, stromal-derived matricellular factors regulate EC sprouting angiogenesis and vascular lumen formation in vitro^18,19^. Taken together, it is clear that stromal cells are critical regulators of capillary network structure and function.

In order to better understand organ-specific features of the capillary endothelium, multicellular models of organotypic vasculature have been developed. 2D in vitro monolayers of organotypic EC are generally simple and inexpensive to create, but typically lack the multicellular complexity (i.e., are grown and studied in the absence of stromal cells) and geometry of the tissue microenvironment. In vivo animal models are perhaps more physiologically relevant, but are often cost- and time-intensive, do not necessarily reflect the human endothelium, and have limited spatiotemporal resolution. We and others have reported on the development of 3D human microvessel networks (both perfused and unperfused) as improved models to understand dynamic events such as angiogenesis^20,21^ and cellular (immune and cancer) trafficking^22–24^. While some effort has been made to validate organ-specific characteristics of the vasculature in these 3D in vitro models^22,25–27^, there is a significant gap in our knowledge of both the similarities and differences that emerge between in vivo and in vitro EC and capillary networks.

We hypothesized that OSC impact capillary EC phenotype, and that 3D capillary networks generate EC that are more representative (relative to 2D monolayer) of in vivo EC. To address our hypothesis, we examined both the EC and OSC transcriptome, using single cell sequencing, in 3D in vitro capillary networks created with OSC representing bone marrow, heart, skin, lung, pancreatic cancer, and normal pancreas. Our results demonstrate that: (1) five distinct EC phenotypes can be defined based on their transcriptome; (2) the relative proportion of each EC phenotype in the 3D networks depends on the OSC; and (3) the proportion of the EC phenotype characterized by active protein synthesis is negatively correlated with key morphological features of actively growing capillary networks including total length and number of junctions. Furthermore, each OSC demonstrated a unique population of cells as well as two populations that were shared amongst the six different OSC. Finally, compared to 2D monolayers of ECFC-ECs, the ECs from the 3D in vitro microvessel networks begin to more closely resemble the in vivo transcriptome from publicly available datasets^28–30^.

## Methods

### 2D monolayer cell culture

Endothelial cell colony-forming endothelial cells (ECFC-ECs) were isolated from umbilical cord blood, as previously described^31,32^, and were chosen as they can be considered “organ-agnostic” (derived from cord blood) and thus represent a less differentiated (progenitor) endothelial cell compared to tissue-resident endothelial cells. The ECFC-ECs were grown on gelatin-coated tissue culture plastic in EGM-2 (Lonza #CC-3162). The same donor of ECFC-ECs was used for all experiments. All organotypic stromal cells (OSCs) were purchased commercially and grown according to vendor recommendations on tissue culture plastic. Human bone marrow stromal cells (Marrow OSC; Lonza #2M-302) were cultured in Myelocult H5100 media (STEMCELL Technologies #05150) supplemented with 1 µM hydrocortisone in a-MEM (STEMCELL Technologies #74142 and ThermoFisher #12571063, respectively), 2 mM l-glutamine (ThermoFisher #25030081), and 50 unit/mL Penicillin–Streptomycin (ThermoFisher #15070063). Human cardiac fibroblasts (Heart OSC; Cell Applications #306AK-05a) were grown in HCF Growth Medium (Cell Applications #316K-500). Normal human dermal fibroblasts (Skin OSC; Lonza #CC-2511) were cultured in FGM-2 (Lonza #CC-3132). Normal human lung fibroblasts (Lung OSC; Lonza #CC-2511) were cultured in FGM-2 (Lonza #CC-3132). Pancreatic cancer associated fibroblasts (cPancreas OSC; Vitro Biopharma #CAF08) were cultured in Pancreatic Stellate CAF Maintenance Media (Vitro Biopharma #PC00B5). Human pancreatic stellate cells (nPancreas OSC; ScienCell Research Laboratories #3830) were cultured in Stellate Cell Complete Medium (ScienCell Research Laboratories #5301).

All cells were sub-cultured per vendor recommendations in a 37 °C, 5% CO_2_ incubator. Once cells reached confluence, they were briefly washed with sterile DPBS without divalent cations (DPBS(−)) (ThermoFisher #14190094) and then treated with 0.05% Trypsin–EDTA (ThermoFisher #25300062). Once lifted, the cell suspension was neutralized using cell-specific media. Cells were then centrifuged at 300×*g* for 5 min and were either (1) propagated for additional sub-culture, (2) utilized in downstream experiments, or (3) frozen down in a 10% DMSO solution for long-term liquid nitrogen storage. ECFC-ECs were used at passage P3–P6 post-cord isolation. Both Lung OSCs and Skin OSCs were used at passage P5–P7. Both Heart OSCs and cPancreas OSCs were used at passage P8–P10. Marrow OSCs were used at passage P2–P4. nPancreas OSCs were used at passage P5–P7.

### Flow cytometry

Once cell monolayers reached confluence, cells were lifted using Trypsin as described above. Cells were blocked in a 0.1% BSA buffer and labeled using conjugated antibodies (Supp. Table 1). Cells were analyzed using a ThermoFisher Attune (model #A24858), with an average of 20,000 events recorded per sample. Gating for all relevant markers of interest was determined using fluorescence-minus-one (FMO) controls for each individual marker of interest. Raw FCS files were processed using FlowJo. Cells were first gated for live, single cells prior to evaluating the expression of markers of interest. Flow cytometry analysis was performed on 3 independent biological replicates of each cell type to verify consistency in marker expression.

### 3D in vitro microvessel networks in fibrin hydrogels

Fibrinogen (Sigma #F8630) was solubilized in DPBS(−) at a concentration of 10 mg/mL, and syringe-filtered prior to use. Thrombin (Sigma #T4648) was solubilized in DPBS(−) at a concentration of 50 units/mL, syringe filtered, and stored at − 20 °C until thawed for use in experiments. Once ECFC-EC and OSC monolayers reached confluence, the cells were exposed to Trypsin and collected as previously described. To create the 3D microvessel networks, we used a well-established previously published protocol^11,33^. In brief, the OSC and ECFC-EC were counted and mixed in sterile fibrinogen at a 2:1 ratio, respectively, such that there were 2 million OSCs per mL and 1 million ECFC-ECs per mL. 150 µL of the cell-fibrinogen solution was mixed with 6 µL of thrombin and the resultant volume introduced into individual wells of a 48 well plate. As a negative control, some fibrin hydrogels contained only 1 million ECFC-ECs per mL (no OSCs present in these hydrogels). The final concentration of fibrinogen in the hydrogels was 10 mg/mL, with a final thrombin concentration of 2 units/mL. The hydrogels were allowed to polymerize for 30 min in a 37 °C incubator prior to adding media. Fibrin hydrogels were cultured in EGM-2, with media changes every other day.

### Immunofluorescence

After 7 days of culture, fibrin hydrogels were fixed in a 10% formalin buffer. Hydrogels were then permeabilized in PBS + 0.5% Tween-20 before undergoing blocking in 2% BSA in PBS + 0.1% Tween-20. Primary CD31 (PECAM1) antibody was diluted in blocking buffer and applied overnight to the hydrogels at 4 °C (Supp. Table 2). Secondary antibody was diluted in blocking buffer and applied for 45 min. at room temperature (Supp. Table 2). Hydrogels were then washed in PBS + 0.1% Tween-20 and imaged. All fibrin hydrogels were imaged using an Olympus IX83 widefield fluorescence microscope. A total of 4–6 replicates were stained for each organ-specific hydrogel condition, and a total of 4–6 fields of view were acquired per replicate using a 10× objective, for a total of 16–36 images for each 3D in vitro microvessel network condition.

### Quantitative evaluation of microvessel networks

Resultant images of the CD31-labeled microvessel networks were edited in ImageJ to further reduce background signal. Resultant JPG files were then quantified using Angiotool, as previously described^33–36^. All images were processed and quantified using the same settings, so as to minimize any error. The resultant data were further analyzed in the R computing environment (see “Statistical analysis” section below). Two parameters were highlighted, namely total vessel length and total number of junctions (or branching points). This workflow is further outlined in Supp. Fig. 2.

### Isolation of cells from fibrin hydrogels

After 7 days of culture, fibrin hydrogels were digested using nattokinase (Japan Bio Science Laboratory #NSK) diluted to 50 units/mL in EGM-2. After 1 h of exposure at 37 °C, resultant degraded hydrogels were pooled for each condition (> 5 hydrogels per condition), washed several times in DPBS(−), and exposed to an additional 10 min of 0.05% Trypsin–EDTA to ensure adequate release of cells from any remnant ECM fragments. Resultant solutions underwent additional washes, 70 µm filtration, and then finally a 40 µm filtration prior to scRNA-Seq analysis.

### Transcriptome alignment and initial processing

All single cell library preps and sequencing was performed by the UC Davis DNA Technologies Core and UC Davis Bioinformatics Core Facilities. Cells were analyzed by 10× Genomics 3′ sequencing v3. Raw fastq files were processed using CellRanger count (10× Genomics) for genome alignment via the Linux command line. Resultant filtered output files were brought into the R computing environment and analyzed further via the Seurat pipeline and by using a series of scRNA-Seq analysis packages (Supp. Table 3). All data files underwent quality control filtering to exclude cells with fewer than 200 unique genes, greater than 7500 unique genes, and/or more than 10% of total gene expression derived from mitochondrial-specific genes (Supp. Fig. 4a–e). In addition, genes that were not detected in at least 3 cells were excluded from downstream analysis. This quality control process yielded several thousand cells per 3D in vitro microvessel network condition (Supp. Fig. 4a). ECs and OSCs were identified by characteristic gene expression, and then separated and re-normalized for downstream analysis. The following values were used for the “resolution” parameter to determine the number of clusters: 1 for the overall 3D in vitro microvessel network-derived cell data (Fig. 2c), 0.3 for the endothelial cells alone from the dataset (Fig. 4a), and 1 for the stromal cells alone from the dataset (Fig. 5a). Clustering was verified by observation and evaluation of the silhouette scores for each dataset (Supp. Fig. 4f,g for overall 3D in vitro microvessel network-derived cell data; Supp. Fig. 6e,f for the endothelial cells alone; Supp. Fig. 7e,f for the stromal cells alone). Additionally, cells were analyzed for their ribosomal gene-related gene expression by analyzing the total amount of *RPS* and *RPL* gene expression per cell, and percentage of *RPS/RPL* gene expression was reported (Supp. Fig. 6g,h for endothelial cells; Supp. Fig. 7g,h for stromal cells).

### Gene ontology analysis

Differentially expressed genes (DEGs) were identified for all EC clusters and OSC clusters (after re-normalizing and re-clustering the data independently). For a specific cluster, the list of DEGs along with the list of all genes expressed was fed into the topGO (v2.42.0) package in R. In order to identify the relevant over-enriched biological process gene ontology (GO) terms associated with the DEGs for each cluster, Fisher’s exact test using the “elim” algorithm was performed. Resultant *p*-values were then log10-transformed and GO terms were rank ordered by the log10-transformed *p*-value.

### In vivo comparisons of in vitro microvessel network data

Organ-matched, publicly available in vivo scRNA-Seq datasets were utilized for comparison to the 3D in vitro microvessel network scRNA-Seq dataset^28–30,37,38^. For all in vivo datasets, ECs were identified out of the larger in vivo datasets by EC characteristic gene expression. Next, the identified in vivo ECs underwent an anchor-based integration with the corresponding organ-specific EC 3D in vitro microvessel network data and ECFC-EC 2D in vitro monolayer data via FindIntegrationAnchors and IntegrateData in Seurat. For each integration, all available in vivo ECs and 3D in vitro microvessel network ECs were utilized. Either 300 or 600 ECFC-ECs from the ECFC-EC 2D in vitro monolayer dataset were also used, so as to match the number of 3D in vitro microvessel network ECs used in the integration. The resultant integrated object was scaled and clustered. DEGs were identified for each EC type (in vivo, 3D in vitro, 2D in vitro monolayer), and then a score was assigned to each EC type based on the expression of the top 20 in vivo DEGs using AddModuleScore in Seurat. The resultant score was then normalized by the mean 3D in vivo score and the mean 2D ECFC-EC in vitro monolayer score, so as to better identify any shift of the 3D in vitro microvessel network-derived ECs relative to the 2D in vitro monolayer and in vivo data.

### Statistical analysis

Statistical analysis of microvessel network data was carried out in the R computing environment. One-way ANOVA was used to compare the total vessel lengths and total number of junctions between the different 3D in vitro microvessel networks (data acquisition outlined in “Immunofluorescence” and “Quantitative evaluation of microvessel networks” sections above). Reported *p*-values were deemed significant if *p* < 0.05. Evaluation of the linear regression EC cluster percentage and mean total vessel length was also performed in R. Pearson’s correlation coefficients were determined as part of the analysis and reported *p*-values were deemed significant if *p* < 0.05.

## Results

### Endothelial cells assemble into microvessel network structures within 7 days when co-cultured with organotypic stromal cells in fibrin hydrogels

Prior to co-culture in a fibrin hydrogel, various OSC monolayers and EC monolayers were examined by flow cytometry to ensure both characteristic EC and OSC marker expression (Supp. Fig. 1a–c). ECFC-ECs were observed to be CD31+CD90−CD144+, consistent with previous observations^39,40^. Crucially, all OSCs were CD31−, but exhibited heterogeneous expression of CD90 and the pericyte marker PDGFRβ.

**Figure 1:**
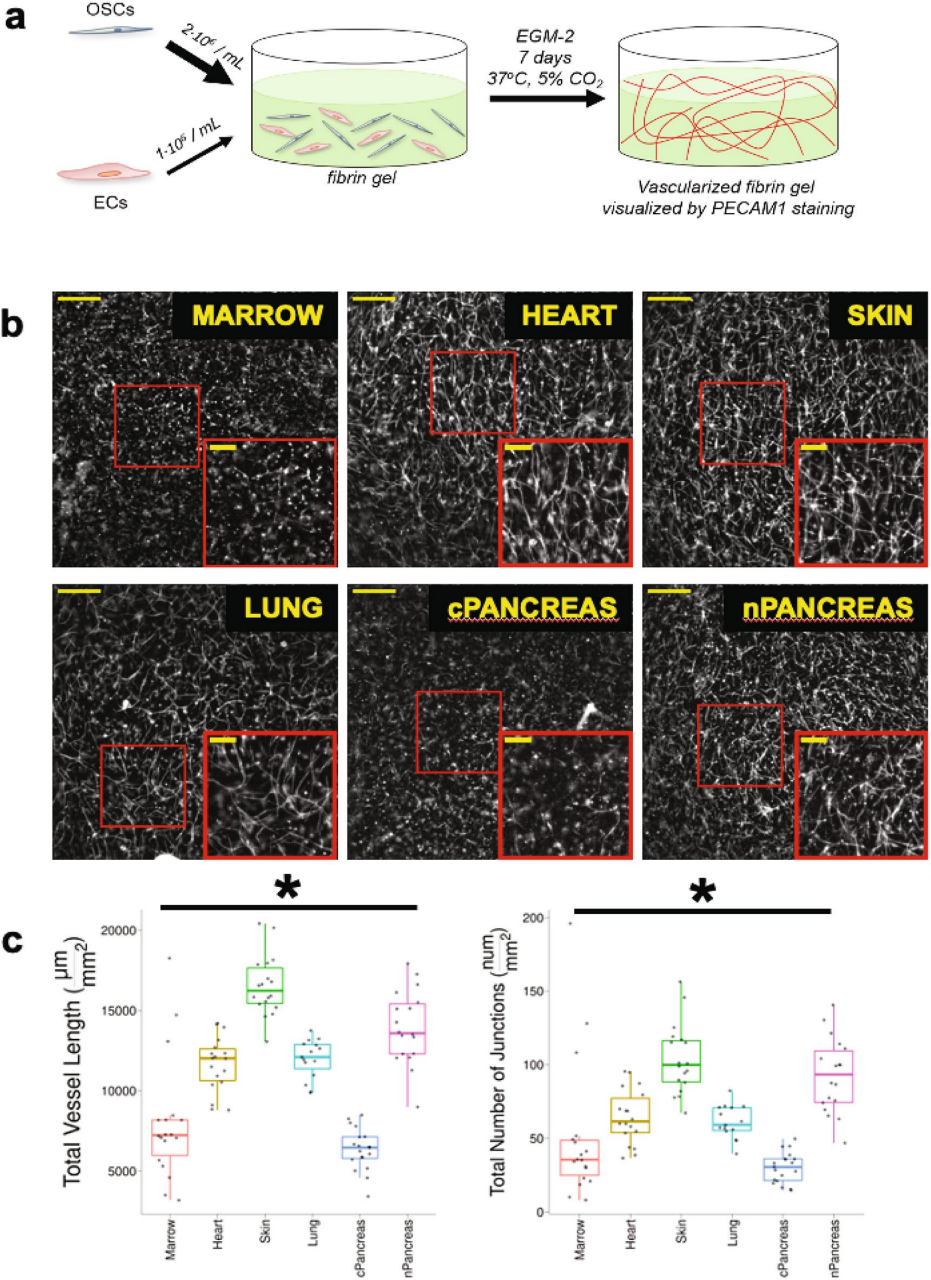
ECs form into CD31 + microvessel networks after 7 days of co-culture with a variety of OSCs in 3D fibrin hydrogels. (**a**) Schematic detailing formation of 3D in vitro fibrin hydrogels and development of microvessel networks. (**b**) Representative images of CD31 + microvessel networks formed using same parental ECFC-EC monolayer and OSCs indicated in each image. Larger image (lower magnification) scale bar represents 500 µm. Inset image is zoomed in image of red-outlined region in lower magnification image and has inset scale bar representing 200 µm. (**c**) Quantification of CD31 + microvessel networks by Angiotool. Total vessel length and total number of junctions normalized by total image area. *p < 0.05 by One-Way ANOVA.

ECFC-ECs assemble into microvessel networks after 7 days of co-culture with each of the six different OSCs in 3D fibrin hydrogels as verified by IF microscopy for CD31/PECAM1 (Fig. 1a,b). The microvessel networks demonstrated statistically significant morphological differences in terms of total vessel length per unit area and total number of branch points per unit area, as determined by One-way ANOVA (*p < 0.05; Fig. 1c). A basic workflow for the quantification of these microvessel networks is provided in the *Methods* above (also Supp. Fig. 2). For example, the total vessel length per unit area for the skin network was approximately twice that of bone marrow or pancreatic cancer. ECs do not form stable microvessel networks in the absence of OSCs in fibrin hydrogels (data not shown). OSCs have previously been shown to be required to support stable 3D in vitro microvessel networks, and also demonstrate pericyte-like behavior and co-localization with microvessels^14,31,41^.

### ECs and OSCs can be identified by characteristic gene expression

**Figure 2:**
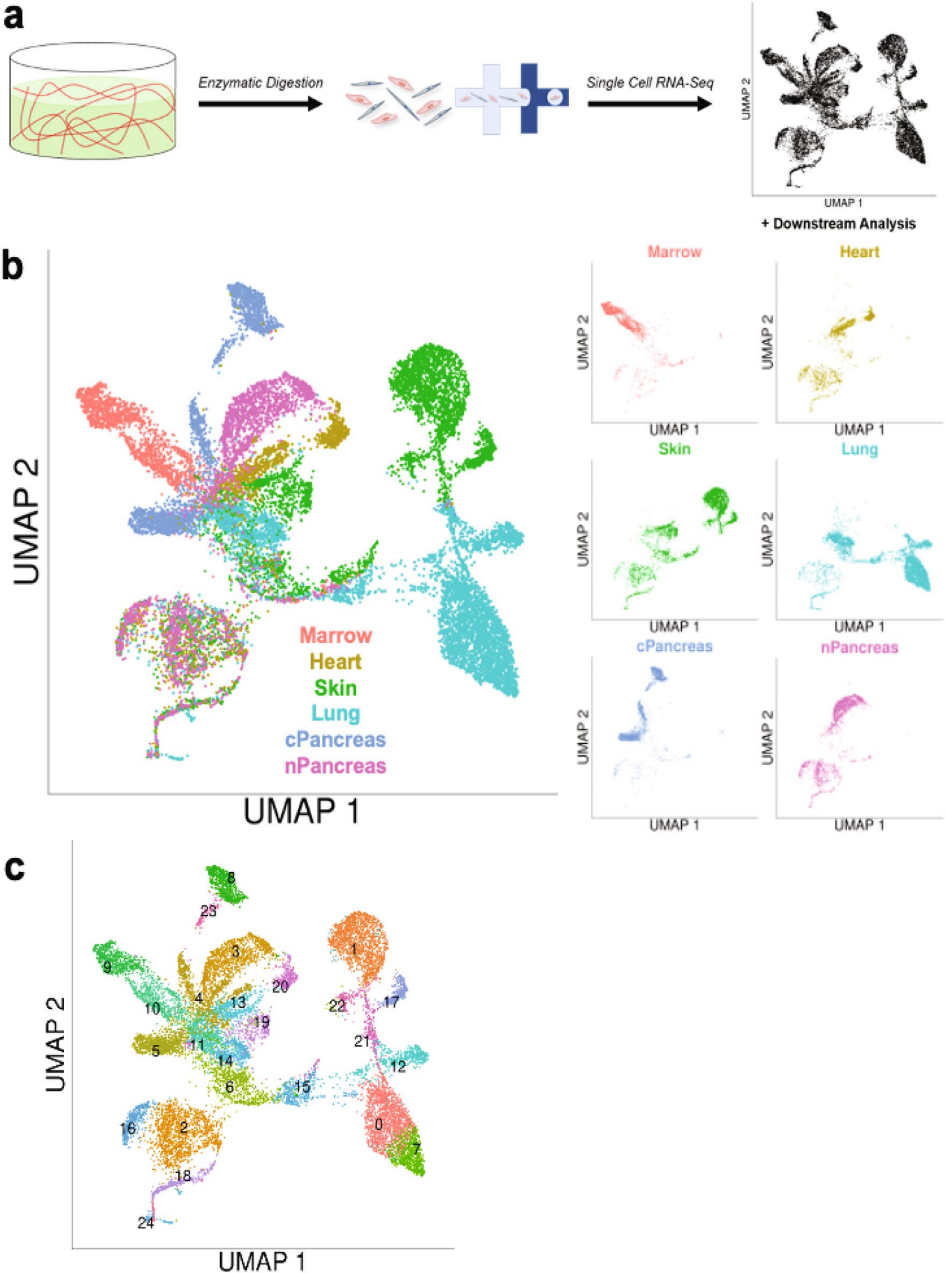
Cells isolated from 3D in vitro fibrin hydrogels can be subjected to scRNA-Seq analysis and are arranged into distinct clusters in UMAP space. (**a**) Schematic detailing processing of 3D in vitro hydrogels for scRNA-Seq. (**b**) Resultant UMAP plots of cells isolated from 3D in vitro fibrin hydrogels comprised of the 6 unique microvessel network types. Additional UMAP plots are broken down by microvessel network type. (**c**) Unsupervised k-means clustering of 3D in vitro hydrogel-derived cells results in 25 distinct clusters.

**Figure 3:**
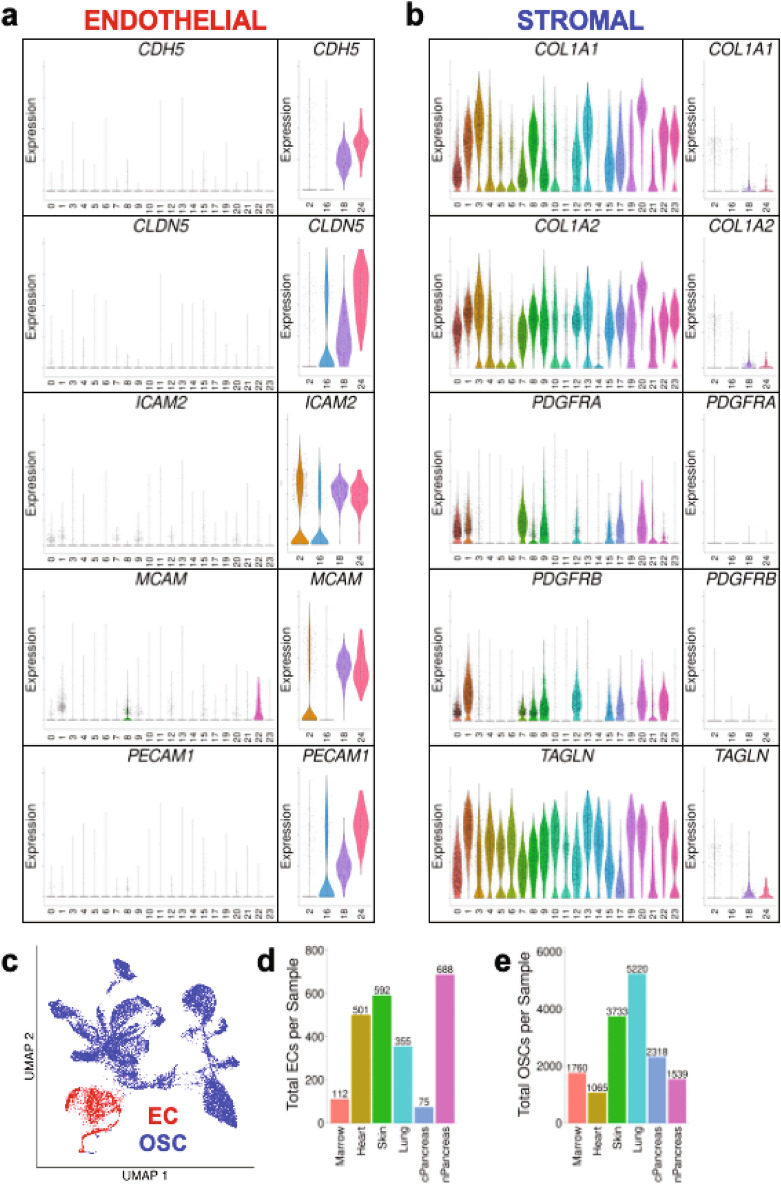
ECs and OSCs can be identified from 3D in vitro microvessel network dataset by expression of characteristic EC-specific and stroma-specific genes. (**a**) Violin plots of characteristic EC genes *CDH5, CLDN5, ICAM2, MCAM,* and *PECAM1* for each cluster identified in complete 3D in vitro microvessel network dataset. (**b**) Violin plots of characteristic stromal cell genes *COL1A1, COL1A2, PDGFRA, PDGFRB,* and *TAGLN* for each cluster identified in complete 3D in vitro fibrin hydrogel dataset. (**c**) Overall UMAP plot for complete 3D in vitro microvessel network dataset broken down by EC/OSC classification (EC = red, OSC = blue). (**d**) Bar chart of resultant number of ECs per microvessel network type from the complete 3D in vitro microvessel network dataset. (**e**) Bar chart of resultant number of OSCs per microvessel network type from the complete 3D in vitro microvessel network dataset.

A protocol to degrade similarly sized fibrin hydrogels was optimized^22,42^, in order to determine the most efficient duration and concentration of fibrinolytic enzyme (Supp. Fig. 3). 3D in vitro fibrin hydrogels then underwent enzymatic digestion (outlined in the “Methods” section), followed by filtration to obtain cells for scRNA-Seq (Fig. 2a). Resultant UMAP plots demonstrate significant transcriptomic heterogeneity both within and between 3D in vitro microvessel network conditions (Fig. 2b). The combined 3D in vitro fibrin hydrogel-derived scRNA-Seq dataset underwent quality control (detailed in the Methods; Supp. Fig. 4a–e) and unsupervised k-means clustering as part of the Seurat pipeline. Parameter sweeps were performed to ensure an appropriate number of clusters was achieved for downstream analysis (Supp. Fig. 4f,g). Twenty-five distinct clusters were identified (Fig. 2c). As previously mentioned, prior to co-culture in the fibrin hydrogels, ECFC-ECs were CD31+CD90−CD144+, while OSCs exhibited heterogeneous expression of CD90 and PDGFRβ, but did not express CD31 as evaluated by flow cytometry (Supp. Fig. 1). Therefore, we proceeded to examine known EC-specific and stromal-specific genes to distinguish the ECs and OSCs in the dataset^18,19,43,44^. Clusters 2, 16, 18, and 24 were notable as they express EC-specific genes including *CDH5*, *CLDN5*, *ICAM2*, *MCAM*, and *PECAM1* (Fig. 3a), albeit with some heterogeneity in marker expression between the clusters. Clusters 2, 16, 18, and 24 also demonstrated heterogeneous expression of other known EC-specific genes (Supp. Fig. 5a)^43^. These four clusters were also negative for a series of stromal-specific genes including *COL1A1*, *COL1A2*, *PDGFRA*, *PDGFRB*, and *TAGLN* (Fig. 3b, Supp. Fig. 5b). The remaining cell clusters (all clusters but 2, 16, 18, and 24) did not express EC-specific genes, while at the same time expressed (with some heterogeneity) stromal-specific genes (Fig. 3a,b, Supp. Fig. 5a,b). We therefore lumped clusters 2, 16, 18, and 24 into a set representing ECs, while all other clusters were considered OSCs (Fig. 3c). This analysis resulted in a range of ECs (75–688) and OSCs (1065–5220) from each of the six microvessel networks (Fig. 3d,e, respectively).

### Distinct EC sub-populations are present in the organotypic microvessel networks

**Figure 4:**
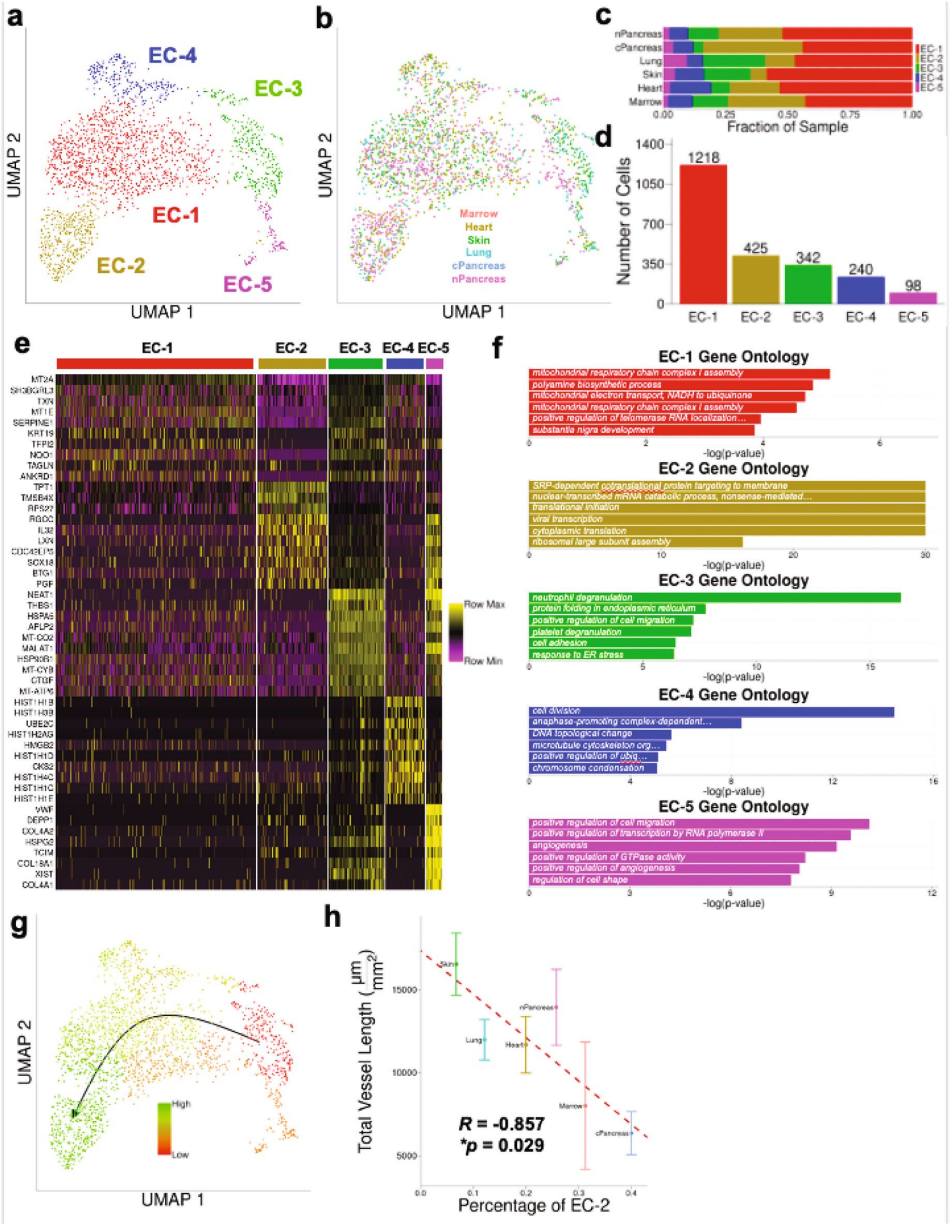
ECs isolated from 3D in vitro microvessel networks separate into distinct clusters with unique transcriptomic profiles. (**a**) UMAP plot of renormalized ECs from the 3D in vitro microvessel network dataset shows ECs separate into 5 distinct clusters (EC-1, EC-2, EC-3, EC-4, and EC-5). (**b**) UMAP plot of renormalized ECs from the 3D in vitro microvessel network dataset grouped by 3D in vitro microvessel network type. (**c**) Grouped bar chart to show relative percentages of each EC cluster per 3D in vitro microvessel network type. (**d**) Bar chart of total number of cells per EC cluster. (**e**) Heatmap of top 10 DEGs for each EC cluster. (**f**) Top 6 most significantly overexpressed GO biological process terms based on top 20 DEGs for each EC cluster. (**g**) Pseudotime values of 3D in vitro EC dataset as determined by Monocle3, mapped onto the UMAP dimensionality reduction of the renormalized ECs (green = late/high pseudotime, red = early/low pseudotime). (**h**) Correlation of the total vessel length measured for each 3D in vitro microvessel network (Fig. 1) compared with the relative percentage of EC-2 in each 3D in vitro microvessel network (**c**). Error bars represent 1 standard deviation above and below the mean total vessel length. Pearson correlation coefficient *R* =  − 0.857 with associated *p* = 0.029.

The total population of ECs was next re-normalized and re-clustered in order to elucidate specific transcriptomic differences between potential sub-populations of ECs. ECs cluster into 5 distinct groups (labeled “EC-1” through “EC-5”), each with distinct transcriptomic profiles (Fig. 4a,b, Supp. Fig. 6). Interestingly, each EC cluster is present in each of the six 3D in vitro organotypic microvessel networks, although the relative fraction of each EC cluster depends on the OSC (Fig. 4c,d). After obtaining a list of differentially expressed genes (DEGs) for each cluster of ECs, we performed gene ontology (GO) analysis to identify distinct biological processes characteristic of each EC cluster (Fig. 4e,f, Supp. Table 4). EC-1 is characterized by DEGs that include redox-related genes (e.g., *MT2A*, *MT1E*, *TXN*), and is associated with enhanced metabolism based on GO terms including “mitochondrial respiratory chain complex I assembly” and “mitochondrial electron transport, NADH to ubiquinone”. EC-2 is characterized by DEGs that include ribosomal-related genes (e.g., *RPS27*) associated with protein regulation and synthesis based on GO terms including “translational initiation” and “ribosomal large subunit assembly”. EC-3 is characterized by DEGs that include cell-ECM regulatory genes (e.g., *THBS1, CTGF*), and is associated with migration and adhesion based on GO terms including “positive regulation of cell migration” and “cell adhesion.” EC-4 is characterized by DEGs that include histone-related genes (e.g., *HIST1H1B*, *HIST1H13B*, *HIST1H2AG*) and cell cycle-related genes (e.g., *UBE2C*, *HMGB2*, *CKS2*), and is associated with cell proliferation based on GO terms including “cell division” and “microtubule cytoskeleton organization.” Finally, EC-5 is characterized by DEGs that include several genes related to endothelial basement membrane (e.g., *COL4A2*, *HSPG2*, *COL4A1*, *COL18A1*), and is associated with angiogenesis based on GO terms including “angiogenesis” and “positive regulation of angiogenesis.” When pseudotime values are mapped onto the UMAP plots (Monocle 3^45^) different EC clusters follow a pattern from a less differentiated EC involved in migration, adhesion, and angiogenesis to a more fully differentiated and synthetic phenotype (EC3 → EC5 → EC4 → EC1 → EC2) (Fig. 4a,g).

In order to better understand the potential impact of EC clusters on the morphology of the individual microvessel networks, we correlated the relative percentage of each EC cluster to the mean total vessel length for each 3D in vitro microvessel network. We observed that the relative fraction of EC-2 (protein synthesis signature) in each microvessel network is negatively correlated with the mean total vessel length per unit area of each 3D in vitro microvessel network (Fig. 4h; *R* = − 0.857, *p = 0.029).

### Common and unique OSC sub-populations are present in 3D organotypic vasculature

**Figure 5:**
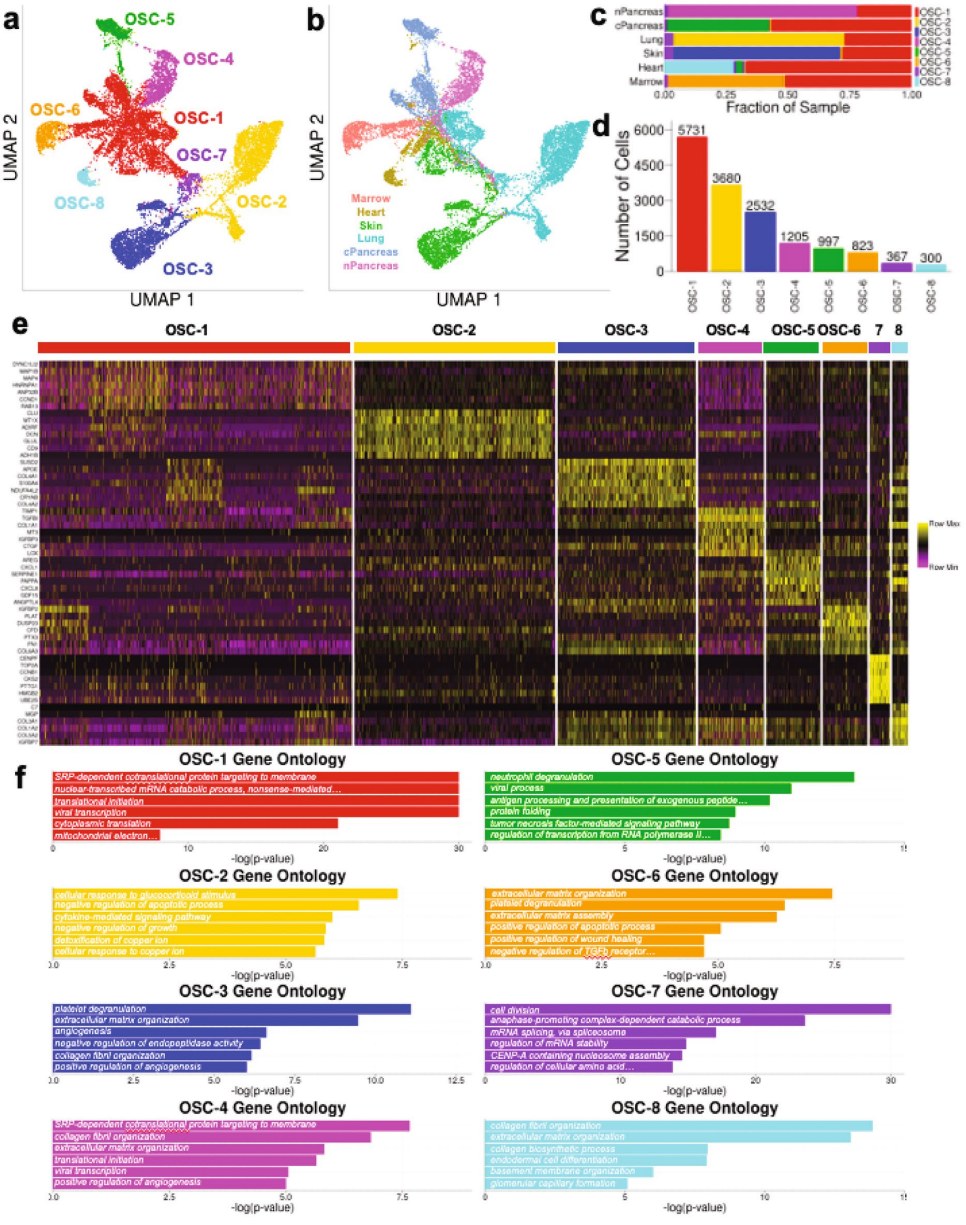
Stromal cells isolated from 3D in vitro microvessel networks separate into distinct clusters with unique transcriptomic profiles. (**a**) UMAP plot of renormalized OSCs from the 3D in vitro microvessel network dataset shows OSCs separate into 8 distinct clusters (OSC-1, OSC-2, OSC-3, OSC-4, OSC-5, OSC-6, OSC-7, and OSC-8). (**b**) UMAP plot of renormalized OSCs from the 3D in vitro microvessel network dataset grouped by 3D in vitro microvessel network type. (**c**) Grouped bar chart to show relative percentages of each OSC cluster per 3D in vitro microvessel network type. (**d**) Bar chart of total number of cells per OSC cluster. (**e**) Heatmap of top 7 DEGs for each OSC cluster. (**f**) Top 6 most significantly overexpressed GO biological process terms based on top 20 DEGs for each OSC cluster.

The population of OSCs was next re-normalized and re-clustered in order to elucidate specific transcriptomic differences between potential sub-populations of OSCs present in the 3D in vitro models of organotypic vasculature. OSCs cluster into 8 distinct groups (labeled “OSC-1” through “OSC-8”), each with distinct transcriptomic profiles (Fig. 5a,b, Supp. Fig. 7, Supp. Table 5). Interestingly, each coculture is represented by a combination of these 8 clusters with each having a unique, dominant OSC cluster. All, however, share 2 clusters—OSC 1 and OSC-7—albeit with the relative fraction of the common OSC clusters being different for each microvessel network (Fig. 5c,d). OSC-2 primarily consists of Lung OSCs; OSC-3 primarily consists of Skin OSCs; OSC-4 primarily consists of nPancreas OSCs; OSC-5 primarily consists of cPancreas OSCs; OSC-6 primarily consists of Marrow OSCs; and OSC-8 primarily consists of Heart OSCs. Thus, each of the OSC populations is transcriptionally distinct from the others, indicating they retain the “memory” of their origin tissue. All of these unique OSC clusters differentially express matricellular genes (e.g., *DCN, LOX, PLAT*) and extracellular matrix proteins (e.g., *COL4A2*, *COL1A1*, *COL6A3*), and have GO terms related to extracellular matrix organization, wound healing, cytokine secretion, and angiogenesis (Fig. 5e,f), reflective of their fibroblast-like identity. OSC-1 is a large population of OSCs present in all cocultures and is relatively poorly defined transcriptionally. OSC-7 is a small population of OSCs present in all microvessel networks and has GO terms related to cell division, suggesting that a relatively small number of OSCs in each coculture are actively proliferating.

### Transcriptomes of 3D in vitro ECs are more similar to in vivo ECs compared to 2D monolayer

We next compared the transcriptome of our 2D EC monolayer and 3D in vitro microvessel networks to publicly available in vivo datasets consisting of cells isolated from human heart, skin, lung, and normal pancreas^28–30^. For each in vivo dataset, we performed k-means clustering, which yielded a different number of clusters for each dataset. The in vivo heart, skin, lung, and normal pancreas datasets yielded a total of 12, 17, 18, and 16 total clusters, respectively. Each in vivo dataset contained a diverse collection of cells for analysis. In each in vivo dataset, we identified ECs by analyzing the differential expression of EC characteristic genes between the different clusters. However, we chose to exclude clusters which appeared to contain lymphatic ECs (by analyzing the expression of lymphatic EC characteristic genes *FLT4*, *LYVE1*, *PDPN*, and *PROX1*)^46,47^.

**Figure 6:**
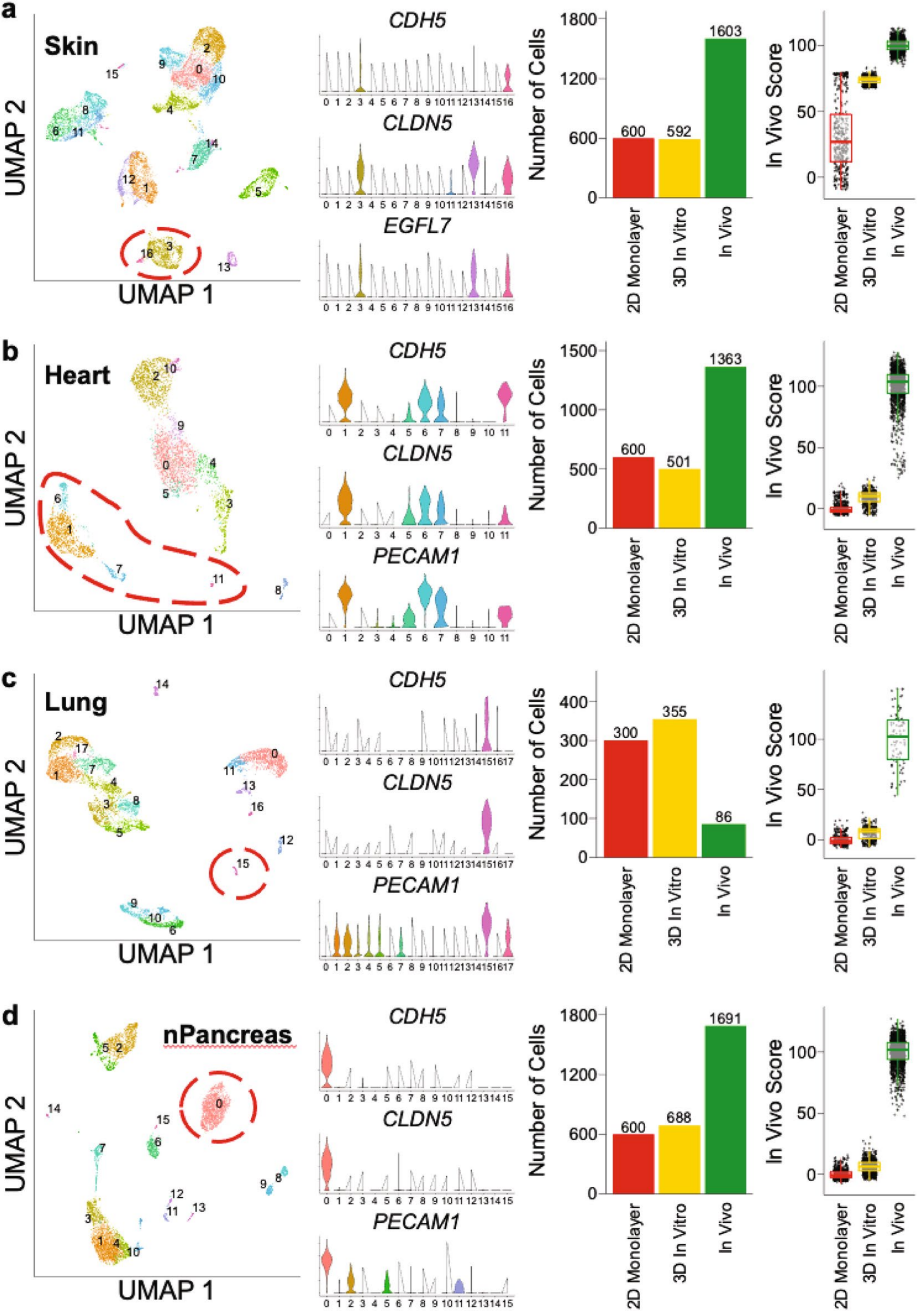
Transcriptomes of 3D in vitro ECs are more similar to in vivo ECs compared to 2D monolayer. (**a**) Comparison of ECFC-EC 2D in vitro monolayer, Heart 3D in vitro microvessel network ECs, and publicly available Heart in vivo ECs. Clustering determined using Seurat, and EC clusters identified using EC characteristic genes *CDH5*, *CLDN5*, and *PECAM1*. In Vivo Score determined by DEGs defining Heart in vivo ECs. *p < 0.05 by One-Way ANOVA. (**b**) Comparison of ECFC-EC 2D in vitro monolayer, Skin 3D in vitro microvessel network ECs, and publicly available Skin in vivo ECs. Clustering determined using Seurat, and EC clusters identified using EC characteristic genes *CDH5*, *CLDN5*, and *EGFL7*. In Vivo Score determined by DEGs defining Skin in vivo ECs. *p < 0.05 by One-Way ANOVA. (**c**) Comparison of ECFC-EC 2D in vitro monolayer, Lung 3D in vitro microvessel network ECs, and publicly available Lung in vivo ECs. Clustering determined using Seurat, and EC clusters identified using EC characteristic genes *CDH5*, *CLDN5*, and *PECAM1*. In Vivo Score determined by DEGs defining Heart in vivo ECs. *p < 0.05 by One-Way ANOVA. (**d**) Comparison of ECFC-EC 2D in vitro monolayer, nPancreas 3D in vitro microvessel network ECs, and publicly available nPancreas in vivo ECs. Clustering determined using Seurat, and EC clusters identified using EC characteristic genes *CDH5*, *CLDN5*, and *PECAM1*. In Vivo Score determined by DEGs defining nPancreas in vivo ECs. *p < 0.05 by One-Way ANOVA.

For the skin in vivo dataset, we identified ECs (clusters 3 and 16) by differential expression of EC characteristic genes *CDH5*, *CLDN5*, and *EGFL7* (Fig. 6a; Supp. Fig. 8a). Importantly, cluster 13 was determined to contain lymphatic ECs due to the expression of lymphatic EC characteristic genes and was excluded from downstream analysis (Supp. Fig. 8). After integrating 2D in vitro monolayer ECFC-ECs, 3D in vitro Skin microvessel network ECs, and in vivo Skin ECs, we assigned a similarity score to each cell in this integrated dataset, based on the top 20 DEGs of the in vivo Skin ECs (further definition of the similarity metric is outlined in the “Methods” section). An identical analysis methodology was used below for heart, lung, and pancreas. The similarity scores for each grouping were statistically different from each other by One-Way ANOVA (*p* < 0.05), where the 2D in vitro monolayer ECFC-EC score was 0 ± 56.47 (mean ± standard deviation); 3D in vitro Skin microvessel network ECs was 73.91 ± 2.97 (mean ± standard deviation); and in vivo Skin ECs was 100 ± 4.54 (mean ± standard deviation). Based on the similarity score, 3D in vitro Skin microvessel network ECs are closer to the in vivo skin ECs, and also demonstrate a much lower inter-cellular variation (less cell to cell heterogeneity in the transcriptome) compared to the 2D in vitro monolayer ECs (*p* < 0.05) (Fig. 6a).

For the heart in vivo dataset, we identified ECs (clusters 1, 6, 7, and 11) by differential expression of EC characteristic genes *CDH5*, *CLDN5*, and *PECAM1* (Fig. 6b). The choice of these clusters was further reinforced by the analysis of other known EC characteristic genes (Supp. Fig. 9a). Additionally, none of these clusters consistently expressed any of the 4 lymphatic genes listed above (Supp. Fig. 9b). The similarity scores for each grouping were statistically different from each other by One-Way ANOVA (*p* < 0.05), where the 2D in vitro monolayer ECFC-EC score was 0 ± 4.64 (mean ± standard deviation); 3D in vitro Heart microvessel network ECs was 8.93 ± 5.56 (mean ± standard deviation); and in vivo Heart ECs was 100 ± 15.80 (mean ± standard deviation).

For the lung in vivo dataset, we identified ECs (cluster 15) by differential expression of EC characteristic genes *CDH5*, *CLDN5*, and *PECAM1* (Fig. 6c). The choice of this cluster was further reinforced by the analysis of other known EC characteristic genes (Supp. Fig. 10a). Additionally, this cluster did not express the 4 lymphatic genes listed above (Supp. Fig. 10b). The similarity scores for each grouping were statistically different from each other by One-Way ANOVA (*p* < 0.05), where the 2D in vitro monolayer ECFC-EC score was 0 ± 5.17 (mean ± standard deviation); 3D in vitro Lung microvessel network ECs was 6.73 ± 6.35 (mean ± standard deviation); and in vivo Lung ECs was 100 ± 26.93 (mean ± standard deviation).

For the normal pancreas (“nPancreas”) in vivo dataset, we identified ECs (cluster 0) by differential expression of EC characteristic genes *CDH5*, *CLDN5*, and *PECAM1* (Fig. 6d). The choice of this cluster was further reinforced by the analysis of other known EC characteristic genes (Supp. Fig. 11a). Additionally, this cluster did not express the 4 lymphatic genes listed above (Supp. Fig. 11b). The similarity scores for each grouping were statistically different from each other by One-Way ANOVA (*p* < 0.05), where the 2D in vitro monolayer ECFC-EC score was 0 ± 3.82 (mean ± standard deviation); 3D in vitro nPancreas microvessel network ECs was 6.38 ± 5.37 (mean ± standard deviation); and in vivo nPancreas ECs was 100 ± 11.09 (mean ± standard deviation). For reference, we also present the top ten differentially expressed genes between the ECFC-EC monolayer, the 3D in vitro microvessel networks (skin, heart, lung, and pancreas), and the in vivo ECs (skin, heart, lung, and pancreas) in Supp. Tables 6–9, respectively.

## Discussion

This study provides new insight into the interplay between stromal cells and endothelial cells at the transcriptomic level in 3D in vitro models of organotypic vasculature. By culturing the same population of organ-agnostic ECs with OSCs, we demonstrate that ECs modulate their transcriptome in response to the specific stromal cell population. The altered EC transcriptome is manifested, in part, by observable phenotypic differences of the microvessel network. Furthermore, the ECs present in a 3D organotypic microvessel network are not transcriptionally homogenous; rather the ECs cluster into five different phenotypes whose relative proportion depends on the OSC. Finally, the presence of the OSC encourages the 3D in vitro microvessel network-derived EC transcriptome to resemble more closely that of the matching organ-specific in vivo ECs compared with the same ECs cultured as a 2D in vitro monolayer.

To generate organ-specific 3D in vitro microvessel networks, we utilized commercially available OSCs and combined these with the same parental donor of ECs (organ-agnostic ECFC-ECs). These ECFC-ECs are isolated from umbilical cord blood (as described in *Methods*) and have been used extensively in in vitro models of the microcirculation by our group and others^20–22,33,41,48–51^. ECFC-ECs have previously been shown to form luminated vascular networks^22,41,51^, respond dynamically to chemical gradients in their local microenvironment^20^, and exhibit marker expression consistent with endothelium^52,53^; thus, they are an appropriate EC to create our organ-specific microvessel networks. By utilizing the same donor source of ECFC-EC for all microvessel networks, we aimed to ensure that any resultant phenotypic and transcriptomic differences observed in the ECs could be attributed solely to the presence of the different OSCs utilized in the hydrogel. The commercially available OSCs readily proliferated on tissue culture plastic in appropriate growth media, and exhibit marker expression consistent with stromal cells (Supp. Fig. 1). Our results demonstrate that OSCs actively proliferate and remodel the ECM while being co-cultured with ECs in the hydrogel with EGM-2 (Fig. 5), which has been previously confirmed by our group^21^.

Interestingly, we observed a significant variation in the number of cells isolated from each microvessel network (Supp. Fig. 3A). This variability may be due to variable efficiency in enzymatic digestion and mechanical disruption for each network. Additionally, there are likely inherent differences in the rate of proliferation of stromal cells between the 3D in vitro microvessel conditions, which may impact cell yield. Finally, variability in cell yield due to extraction techniques (i.e. enzymatic digestion, mechanical dissociation, etc.) is not unique to this study, and has long been recognized when sourcing tissue-derived cells for scRNA-Seq^54^.

Following our initial unsupervised analysis of the transcriptome of all cells, we identified four clusters as endothelial cells (clusters 2, 16, 18, 24). However, of note was the significant heterogeneity in the expression of twelve known endothelial cell genes (Fig. 3a, Supp. Fig. 4) amongst these four clusters. The EC clusters were all positive for some (but not all) characteristic endothelial cell genes; but were also identified as EC by the absence of gene expression (or minimal expression) of a panel of twelve stromal cell-specific genes. Of particular note was our observation that CD31 (*PECAM1*) expression in cluster 2 was essentially undetectable despite being present by flow cytometry in 2D culture prior to 3D microvessel network formation. This could be attributed to the sequencing depth of our analysis (i.e., *PECAM1* may be expressed in the cluster, just at a lower level than other EC clusters, but higher than stromal cells), or may reflect differences in gene (single cell sequencing) and protein (flow cytometry) expression. Alternatively, quiescent EC, with stable intercellular junctions may only require a low level of CD31 expression to maintain those junctions, whereas more actively dividing and migrating cells would require a higher level of turnover.

After re-normalizing and re-clustering only the ECs from the 3D in vitro microvessel network dataset, we observed five distinct clusters (Fig. 4). Four of these clusters (EC-2 through EC-5) demonstrated distinct transcriptomic profiles as shown by the relative overexpression of a small number (8–10) of genes (Fig. 4e). In contrast, EC-1, the largest EC cluster in each of the six cocultures (representing approximately 50% of the cells), is not well-defined; as such, cluster EC-1 may represent a population of EC performing generic EC functions that are common to all vasculature. Cluster EC-2 is negatively correlated with microvessel network length and, based on GO and pseudotime analysis, is consistent with a more stable and differentiated population of ECs involved in active protein synthesis. The negative correlation with microvessel network length may suggest that active microvessel elongation is associated more with migratory processes as opposed to protein synthesis.

Since we only analyzed a single timepoint (7 days), it is possible that the organotypic microvessel networks develop at different rates, and different proportions of the EC clusters would be observed at different timepoints. Cluster EC-5 is the smallest cluster, generally representing < 10% of the ECs in any of the organotypic microvessel networks. It is well-defined, and is characterized by genes associated with angiogenesis, in particular extracellular matrix proteins (e.g., *COL4A2*, *COL18A1*, and *COL4A1*). The presence of clusters EC-4 and EC-5 suggests that 7 days may not be adequate to achieve a truly quiescent microvessel network, or that the presence of growth factors in our media allows these phenotypes to persist.

Perhaps not surprisingly (given that each microvessel network was formed with a specific OSC), each 3D organotypic microvessel network was associated with a population of OSCs characterized by a unique set of DEGs (e.g., OSC-3 and skin OSCs). However, surprisingly this unique cluster in each coculture was associated with GO terms consistent with extracellular matrix production and organization (with notable exception of OSC-5, the only cancer associated fibroblast)—processes generally considered to be shared across stromal cells. As such, these subpopulations of stromal cells may contribute to the morphological differences of the organotypic microvessel networks (Fig. 1b,c), and differences in the EC transcriptome (Fig. 3c). Furthermore, this observation suggests that each stromal cell may invoke unique gene networks to create and support organ-specific extracellular matrix. The stromal cell clusters common to all of the organotypic microvessel networks (OSC-1 and OSC-7) are associated with GO terms related to protein synthesis (OSC-1) and cell proliferation (OSC-7). OSC-1 is the largest stromal cell population in each network (~ 25–60% of stromal cell population), but, as was the case with the largest EC cluster, is not well-defined by a unique set of genes. Thus, OSC-1 may represent a stromal cell population carrying out more generic stromal cell functions that are not unique to a specific tissue.

This study raises important questions regarding how closely 3D in vitro microvessel networks resemble in vivo conditions. While a wide range of OSCs support microvessel network growth in this model system, the mere presence of an OSC does not fully convert, at the transcriptional level, an otherwise naïve EC into an organ-specific EC. While we were only able to compare the transcriptome of our organotypic 3D microvessel networks to four in vivo EC datasets (skin, heart, lung, and pancreas), our results suggest that the EC in the 3D organotypic microvessel network is more similar to the in vivo EC compared to 2D ECFC-EC monolayers cultured alone, but that the degree of similarity may depend on the organ and OSC. The emergence of publicly accessible human organ-specific transcriptomic datasets is a welcome development over the past several years^28–30,55–59^; however, these datasets remain incomplete and do not yet paint a full picture of the EC transcriptome. There are numerous murine datasets^60,61^ which aim to fill in these gaps, but known differences in murine and human EC biology limit their application.

Several limitations of the 3D organotypic microvessel network model system likely contribute to transcriptomic differences with in vivo ECs. First, the model system utilizes fibrin as the ECM basis for the hydrogel. Moreover, the model that we present here utilizes bovine fibrinogen, which differs slightly from human fibrinogen due to subtle amino acid residue insertions at the N-terminus of two of the three constituent polypeptide chains^62,63^. While these differences may impact mechanical and chemical aspects of the hydrogel, it does not appear to negatively affect either the ECFC-ECs nor the OSCs in terms of their respective survival, proliferation, marker expression, or gene expression. These vascularized hydrogel systems are very amenable to the utilization of different types of ECM, including different types and amounts of collagen^35,64,65^, as well. Additionally, the model is supported entirely through diffusion of nutrients through the tissue (no convective flow). It is well known that intraluminal physiologic shear impacts EC phenotype^66,67^, and interstitial shear can also impact both endothelial and stromal cell phenotype^20,68^. The media chosen to support these cells (EGM-2) also contains VEGF and FGF (among other factors), which have both been shown to encourage EC proliferation, migration, and angiogenic sprouting^20,69–71^. Therefore, the 3D in vitro microvessel networks may not be fully quiescent relative to the in vivo capillary bed. Additionally, other common (e.g., immune) and organ-specific (e.g., keratinocyte, cardiomyocyte) cell types are abundant in the interstitium, and are not included in our simple 3D system. These cells are capable of impacting the mechanical microenvironment and/or secreting soluble mediators that could impact vascular network formation and stability^72–74^.

In summary, we created organotypic 3D microvessel networks by combining an organ-agnostic EC with six different OSCs. All six OSCs supported microvessel network formation, suggesting that this simple model system may be a useful and more physiologically relevant model system to investigate processes such as organ-specific angiogenesis. After 7 days, the transcriptome of the ECs in the microvessel networks could be characterized by five different populations, and the relative proportion of each was dependent on the OSC. Furthermore, morphologic features of the microvessel networks, such as total vessel length, correlated strongly with an EC cluster associated with protein synthesis. Each of the OSCs were associated with a unique cluster of cells who transcriptome was associated with extracellular matrix production and organization, suggesting that these processes, common to all organs, may have organ-specific gene pathways. Finally, while the transcriptome of the ECs in the 3D organotypic microvessel networks more closely resembled the transcriptome of in vivo ECs compared to EC in 2D monolayer, there remains a significant gap, which is likely related to factors (cells, mediators, mechanical environment) in the interstitium not present in our model system known to impact EC function.

## Supplementary Information

Supplementary Information.

## Data Availability

3D in vitro microvessel network raw data and 2D in vitro ECFC-EC monolayer data have been made publicly available at NIH GEO (GSE206256).

## Abbreviations

BSA: Bovine serum albumin
cPancreas: Cancer pancreas
DEGs: Differentially expressed genes
DPBS: Dulbecco’s phosphate buffered saline
EC: Endothelial cell
ECFC-EC: Endothelial colony forming cell-endothelial cell
ECM: Extracellular matrix
GO: Gene ontology
nPancreas: Normal pancreas
OSC: Organotypic stromal cell
PBS: Phosphate buffered saline
scRNA-Seq: Single cell RNA-sequencing
UMAP: Uniform manifold approximation and projection
