# Supplementary material for: Organotypic stromal cells impact endothelial cell transcriptome in 3D microvessel networks
Source: Sci Rep. 2022 Nov 28;12:20434. doi: 10.1038/s41598-022-24013-y (PMC9705391; doi:10.1038/s41598-022-24013-y)
Supplement: Supplementary file 1 — Supplementary Information. [file 41598_2022_24013_MOESM1_ESM.pdf]

SUPPLEMENTARY INFORMATION

Supplementary figures (Supp Figs 1-11) are provided followed by supplementary tables (Supp Tables 1-9).

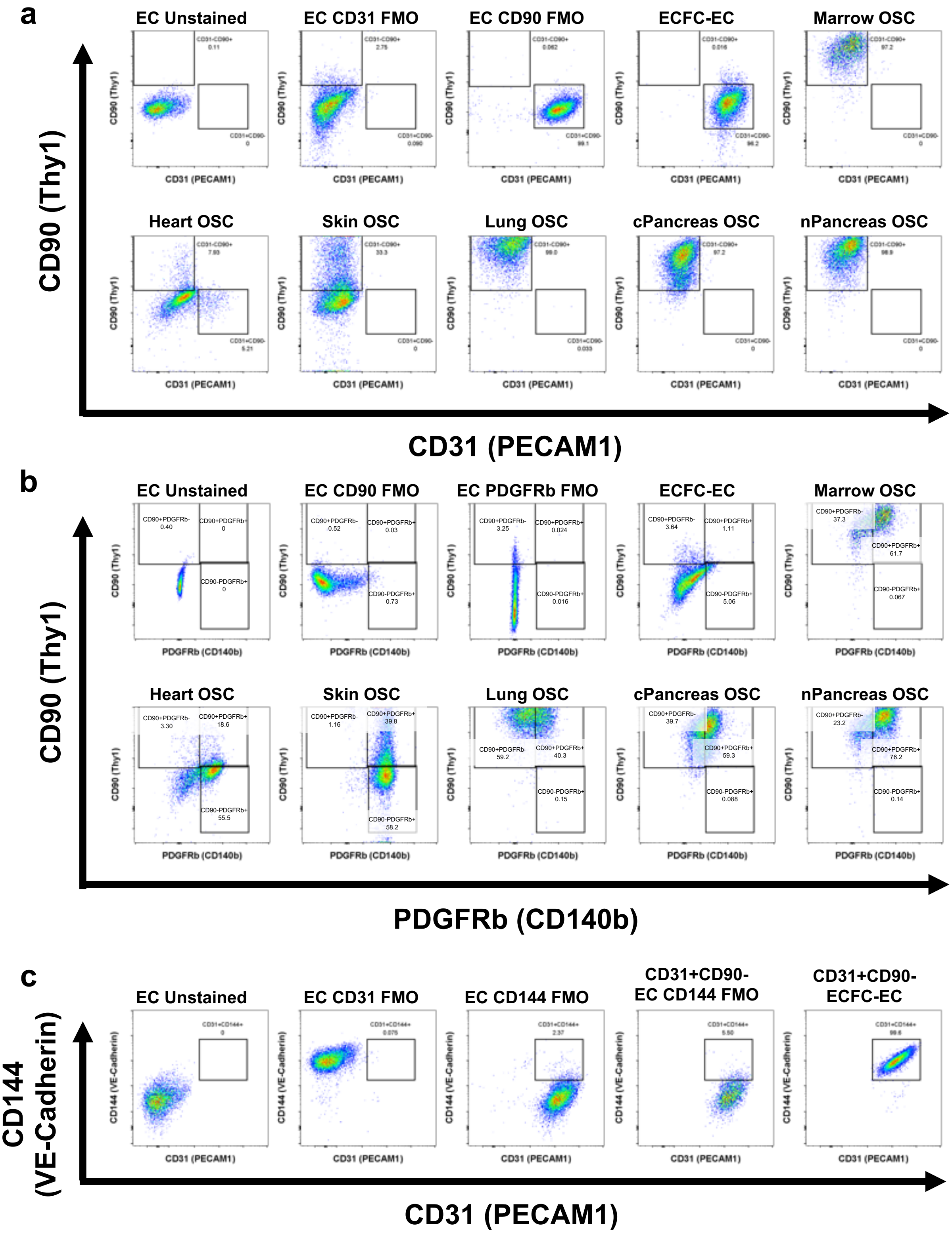
a
EC Unstained
EC CD31 FMO
EC CD90 FMO
ECFC-EC
Marrow OSC
Heart OSC
Skin OSC
Lung OSC
cPancreas OSC
nPancreas OSC
CD90 (Thy1)
CD31 (PECAM1)
b
EC Unstained
EC CD90 FMO
EC PDGFRb FMO
ECFC-EC
Marrow OSC
Heart OSC
Skin OSC
Lung OSC
cPancreas OSC
nPancreas OSC
CD90 (Thy1)
PDGFRb (CD140b)
c
EC Unstained
EC CD31 FMO
EC CD144 FMO
CD31+CD90-
EC CD144 FMO
CD31+CD90-
ECFC-EC
CD144
(VE-Cadherin)
CD31 (PECAM1)

Supp Fig 1 ECFC-EC monolayers are CD31+CD90-CD144+ by flow cytometry, while OSC monolayers have heterogeneous expression of CD90, but crucially do not express CD31. Additionally, some OSC monolayers also exhibit heterogeneous expression of pericyte marker PDGFRb. **a** CD31 vs. CD90 expression for ECFC-EC and OSC monolayers. Representative plots shown from 3 independent experiments of live, single cells. Average of 20,000 events recorded per cell type. Gating determined by FMO controls on EC monolayers. **b** CD31 vs. PDGFRb expression for ECFC-EC and OSC monolayers. Representative plots shown from 3 independent experiments of live, single cells. Average of 20,000 events recorded. Gating determined by FMO controls on EC monolayers. **c** CD31 vs. CD144 expression for ECFC-EC monolayers. Representative plots shown from 3 independent experiments of live, single cells. Average of 20,000 events recorded per image. Gating determined by FMO controls on ECFC-EC monolayers. CD31/CD144 expression also validated on previously gated CD31+CD90- ECFC-EC monolayers where indicated.

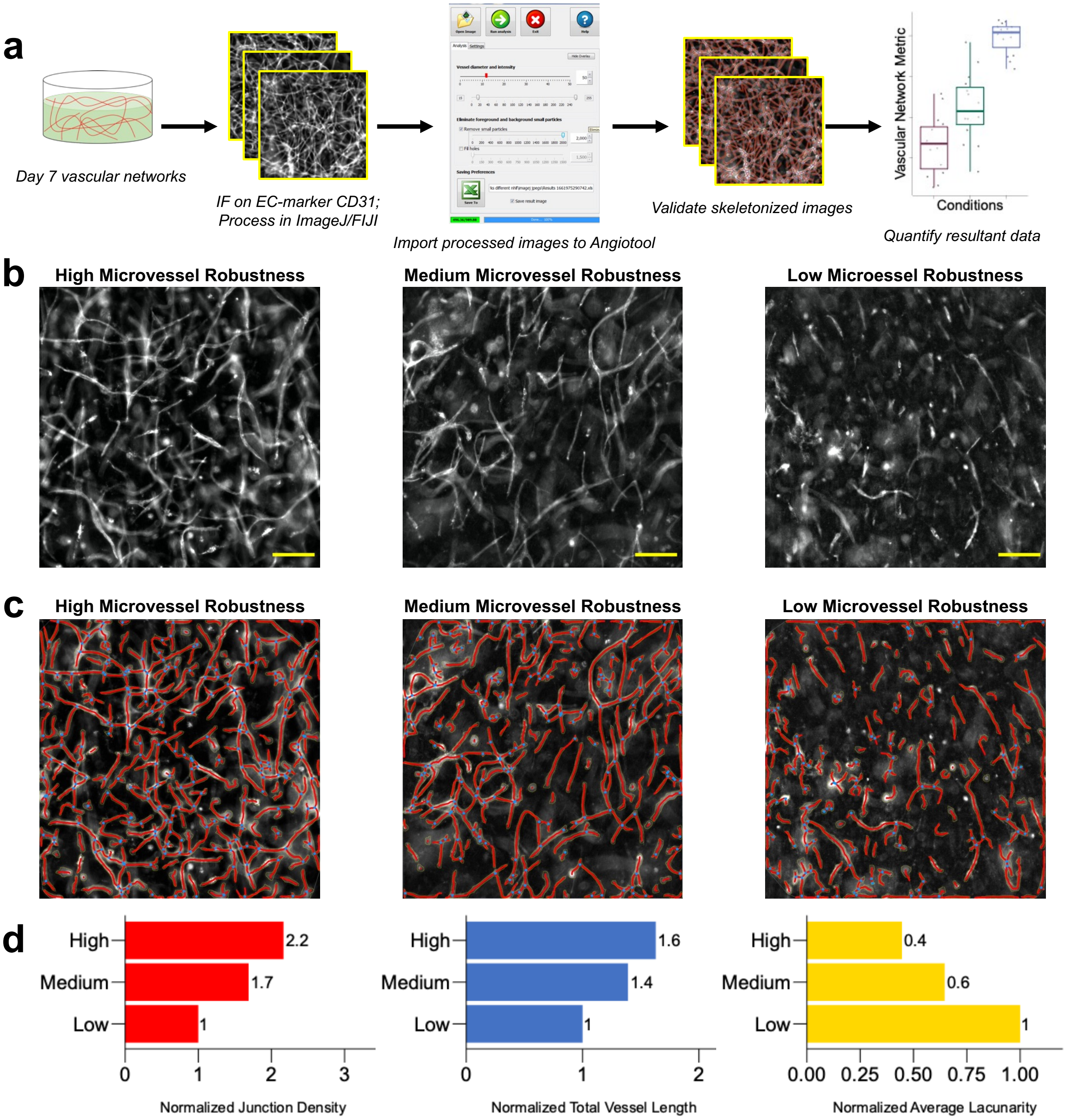
a
Day 7 vascular networks
IF on EC-marker CD31;
Process in ImageJ/FIJI
Import processed images to Angiotool
Validate skeletonized images
Vascular Network Metric
Conditions
Quantify resultant data
b
High Microvessel Robustness
Medium Microvessel Robustness
Low Microessel Robustness
c
High Microvessel Robustness
Medium Microvessel Robustness
Low Microvessel Robustness
d
High
Medium
Low
2.2
1.7
1
0
1
2
3
Normalized Junction Density
High
Medium
Low
1.6
1.4
1
0
1
2
Normalized Total Vessel Length
High
Medium
Low
0.4
0.6
1
0.00
0.25
0.50
0.75
1.00
Normalized Average Lacunarity

Supp Fig 2 Workflow for quantification of microvessel networks using Angiotool. **a** Detailed workflow of microvessel network quantification. Day 7 CD31+ microvessel networks undergo IF microscopy, and resultant images are processed using ImageJ. Processed images are then quantified using Angiotool, which yields skeletonized images and allows for statistical comparison of different microvessel network conditions. **b** Day 7 CD31+ microvessel networks from 3D *in vitro* fibrin hydrogels utilized in this study studies exhibit qualitative differences in vessel robustness. Scale bar represents 200 μm. Each image represents an independent biological replicate (*n* = 1 per vessel density condition). **c** Skeletonized images of the different 3D *in vitro* microvessel networks allows for the quantification of differences between 3D *in vitro* microvessel networks. **d** Resultant quantification of microvessel networks. Junction density, total vessel length, and average lacunarity values normalized by the "Low Microvessel Robustness" condition.

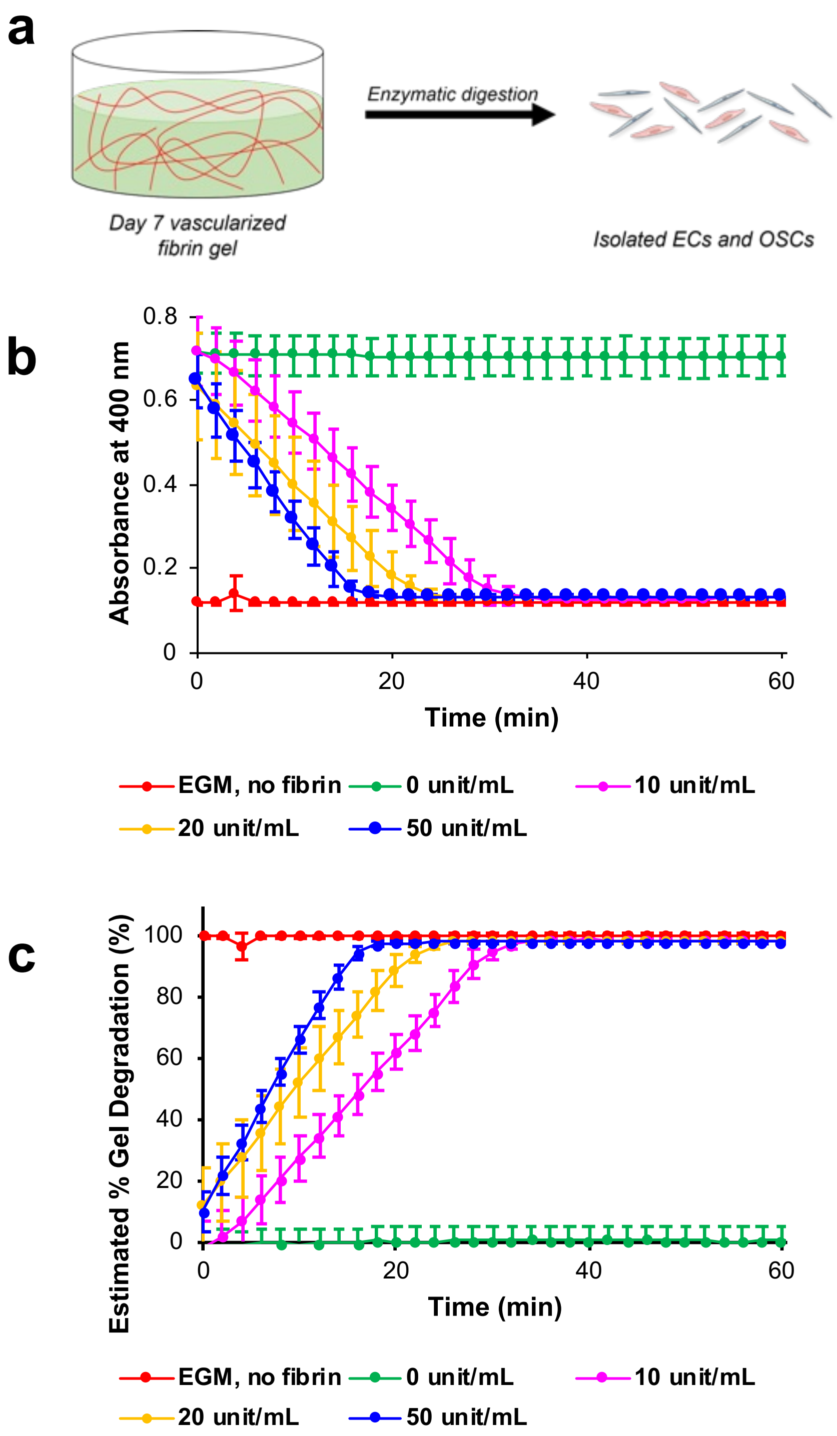
a
Enzymatic digestion
Day 7 vascularized fibrin gel
Isolated ECs and OSCs
b
Absorbance at 400 nm
0.8
0.6
0.4
0.2
0
0
20
40
60
Time (min)
EGM, no fibrin
0 unit/mL
10 unit/mL
20 unit/mL
50 unit/mL
c
Estimated % Gel Degradation (%)
100
80
60
40
20
0
0
20
40
60
Time (min)
EGM, no fibrin
0 unit/mL
10 unit/mL
20 unit/mL
50 unit/mL

Supp Fig 3 3D fibrin hydrogels can be degraded by leveraging fibrinolytic activity of nattokinase. **a** Schematic detailing enzymatic digestion of 3D *in vitro* fibrin hydrogels containing Day 7 microvessel networks consisting of ECs and OSCs. **b** Absorbance values at 400 nm for 150 µL 3D *in vitro* blank hydrogels (fibrin only, no cells) exposed to a variety of concentrations of nattokinase diluted in EGM-2 over the course of 2 hours. Error bars represent 1 standard deviation around the mean absorbance value. Sample size of 3 for each measurement. **c** Estimated percent gel degradation for 150 µL 3D *in vitro* blank hydrogels based on normalization of absorbance values from Supp. Fig. 2B.

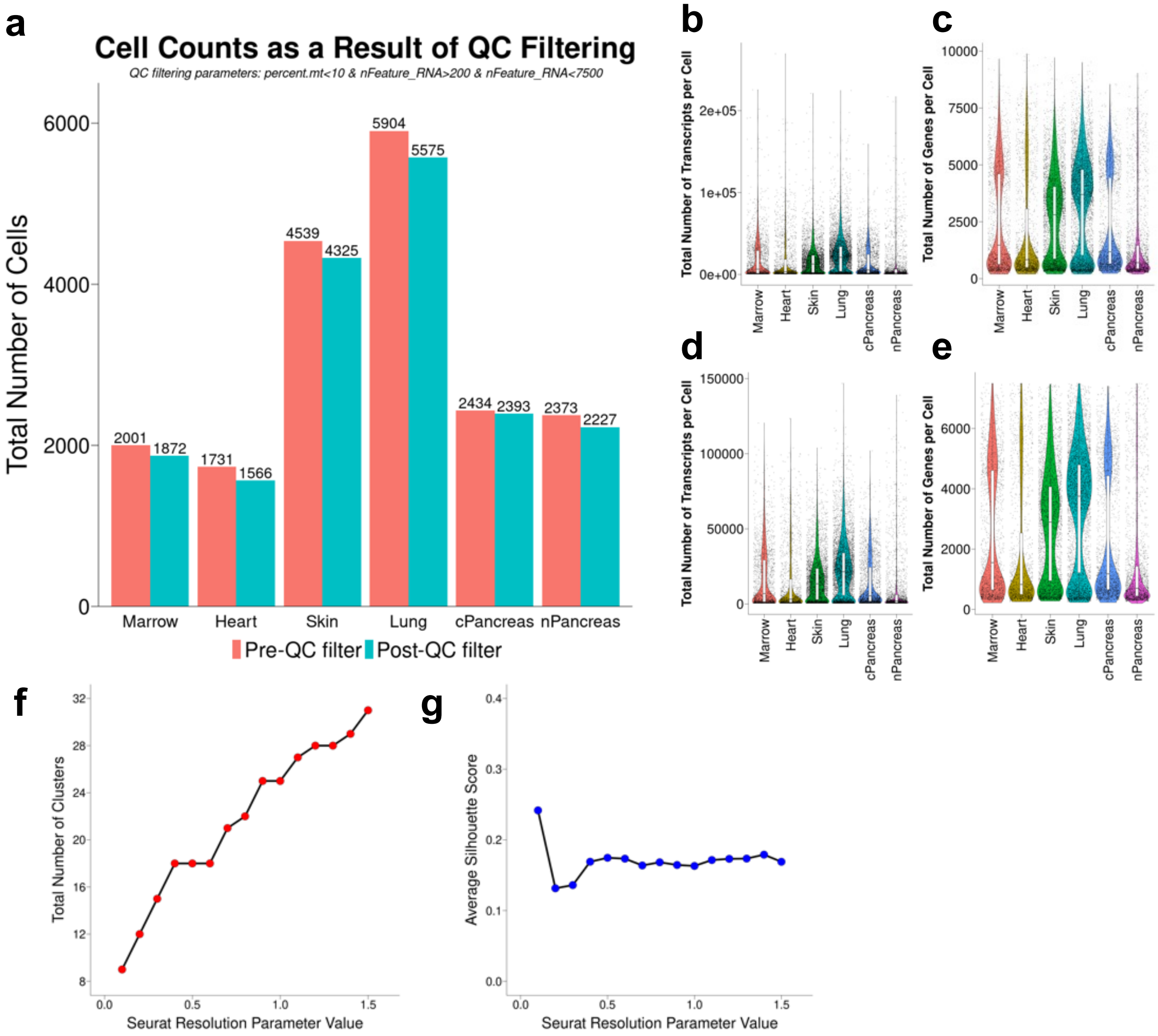
a
Cell Counts as a Result of QC Filtering
QC filtering parameters: percent.mt<10 & nFeature_RNA>200 & nFeature_RNA<7500
Total Number of Cells
2001 1872 1731 1566 4539 4325 5904 5575 2434 2393 2373 2227
Marrow Heart Skin Lung cPancreas nPancreas
Pre-QC filter Post-QC filter
b
Total Number of Transcripts per Cell
c
Total Number of Genes per Cell
d
Total Number of Transcripts per Cell
e
Total Number of Genes per Cell
f
Total Number of Clusters
Seurat Resolution Parameter Value
g
Average Silhouette Score
Seurat Resolution Parameter Value

Supp Fig 4 Quality control of complete 3D *in vitro* microvessel network scRNA-Seq dataset, along with validation of clustering. **a** Bar chart indicating number of cells isolated from each 3D *in vitro* microvessel network condition before and after QC filtering as part of the scRNA-Seq analysis. **b** Violin plot of total number of transcripts for each microvessel network sample before QC filtering. **c** Violin plot of total number of unique genes for each sample before QC filtering. **d** Violin plot of total number of transcripts for each sample after QC filtering. **e** Violin plot of total number of unique genes for each sample after QC filtering. **f** Scatterplot of resultant number of cell clusters to Seurat resolution parameter value (values between 0.1-1.5). **g** Scatterplot of average silhouette score for clusters as determined by Seurat resolution parameter (values between 0.1-1.5).

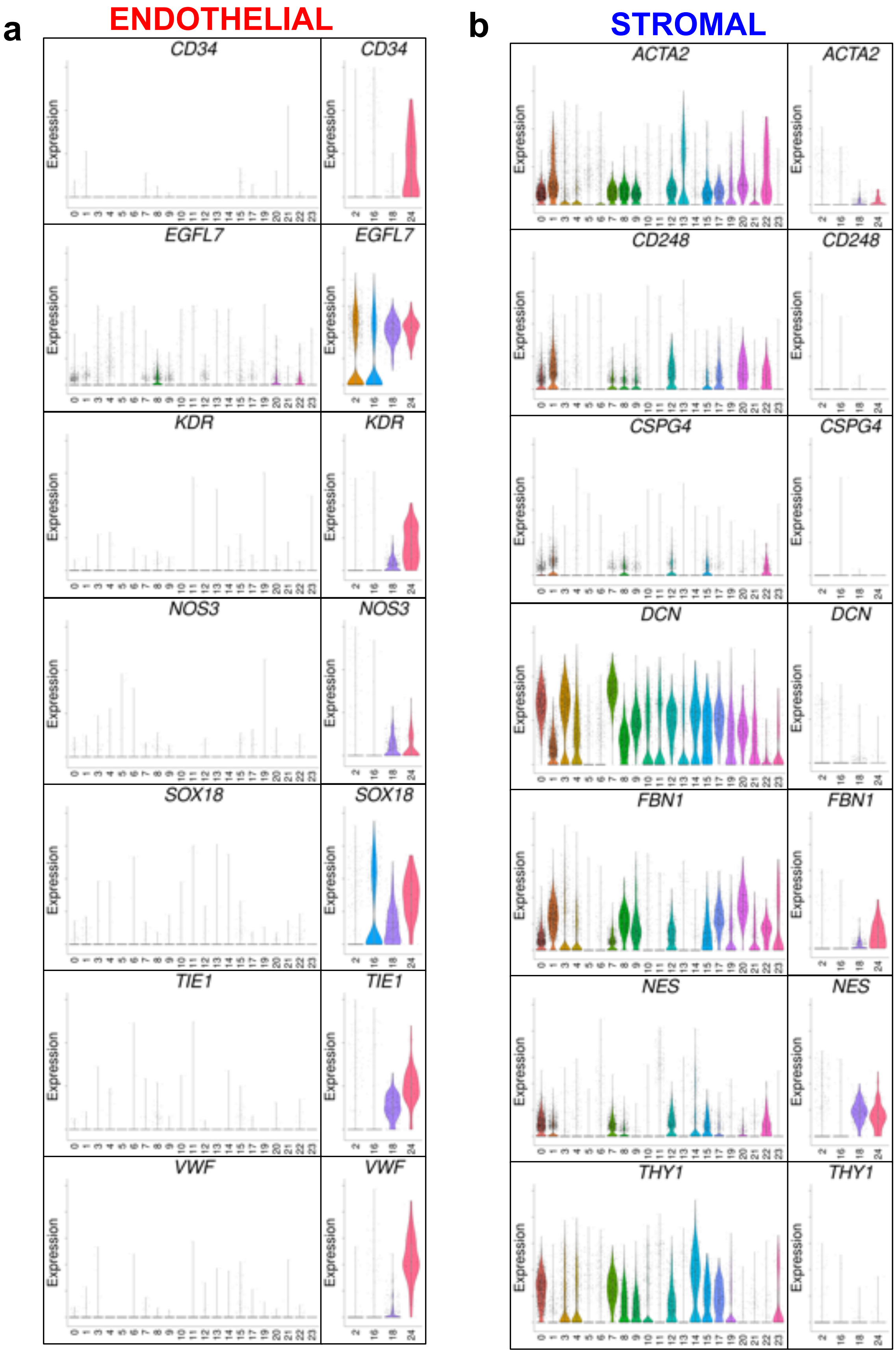
a
ENDOTHELIAL
CD34
EGFL7
KDR
NOS3
SOX18
TIE1
VWF
Expression
b
STROMAL
ACTA2
CD248
CSPG4
DCN
FBN1
NES
THY1
Expression

Supp Fig 5 Additional characteristic EC and OSC gene expression of clusters from 3D *in vitro* microvessel network dataset. **a** Violin plots of additional EC-characteristic genes *CD34, EGFL7, KDR, NOS3, SOX18, TIE1,* and *VWF* for each cluster identified in complete 3D *in vitro* microvessel network dataset. **b** Violin plots of additional stroma-characteristic genes *ACTA2, CD248, CSPG4, DCN, FBN1, NES,* and *THY1* for each cluster identified in complete 3D *in vitro* microvessel network dataset.

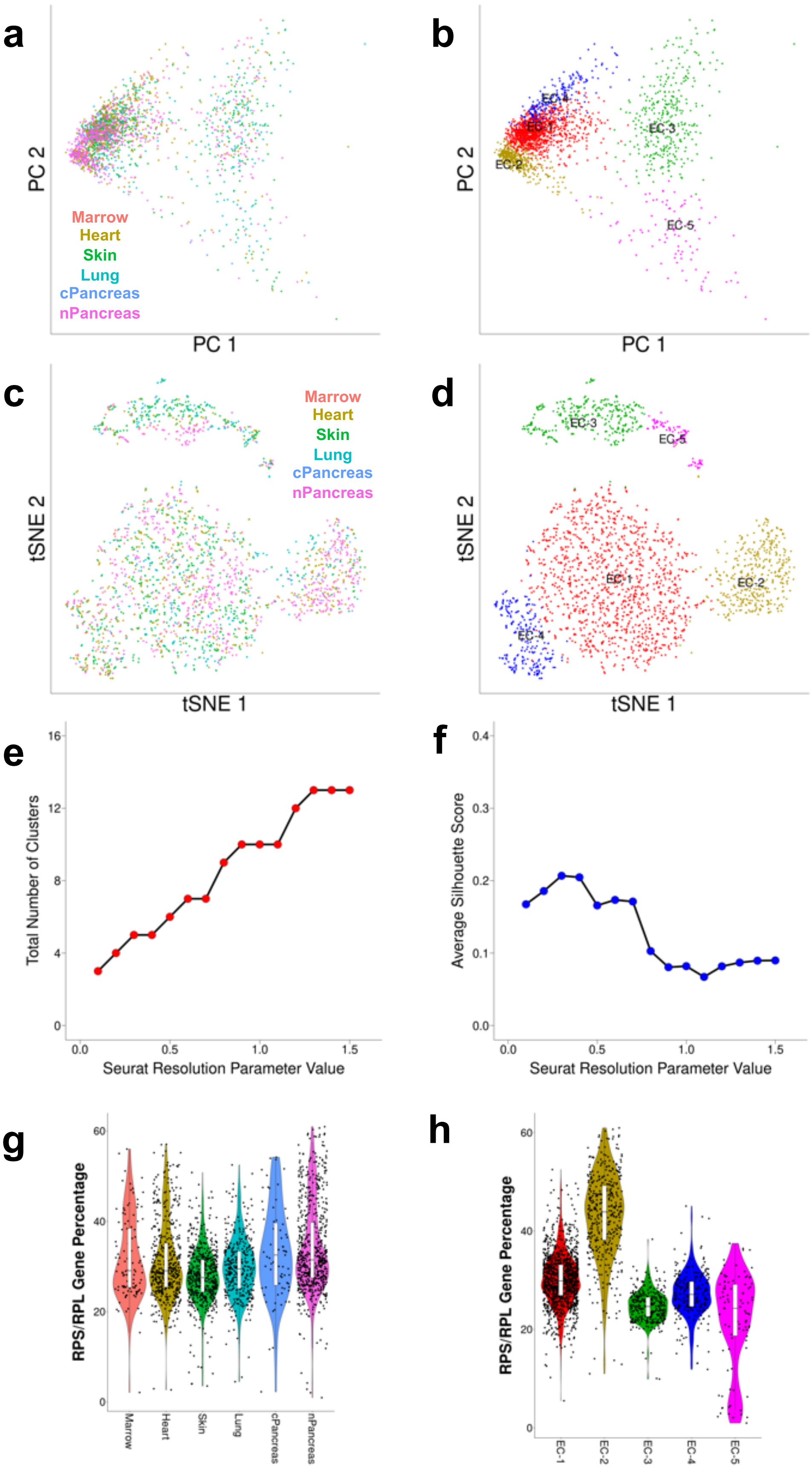
a
b
c
d
e
f
g
h
PC 2
PC 1
Marrow
Heart
Skin
Lung
cPancreas
nPancreas
EC-1
EC-2
EC-3
EC-4
EC-5
tSNE 2
tSNE 1
Total Number of Clusters
Average Silhouette Score
Seurat Resolution Parameter Value
RPS/RPL Gene Percentage

Supp Fig 6 Dimensionality reduction and analysis of clustering results of re-normalized and re-clustered ECs isolated from 3D *in vitro* microvessel network dataset. **a** PCA plot of EC dataset separated by microvessel network type (Marrow, Heart, Skin, Lung, cPancreas, nPancreas). **b** PCA plot of EC dataset separated by cluster identity (EC-1, EC-2, EC-3, EC-4, EC-5). **c** tSNE plot of EC dataset separated by microvessel network type. **d** tSNE plot of EC dataset separated by cluster identity. **e** Scatterplot of resultant number of cell clusters to Seurat resolution parameter value (values between 0.1-1.5). **f** Scatterplot of average silhouette score for clusters as determined by Seurat resolution parameter (values between 0.1-1.5). **g** Violin plot of percentage of ribosomal genes (percentage of gene expression by *RPS* and *RPL* genes per cell) grouped by microvessel network type. **h** Violin plot of percentage of ribosomal genes (percentage of gene expression by *RPS* and *RPL* genes per cell) grouped by EC cluster identity.

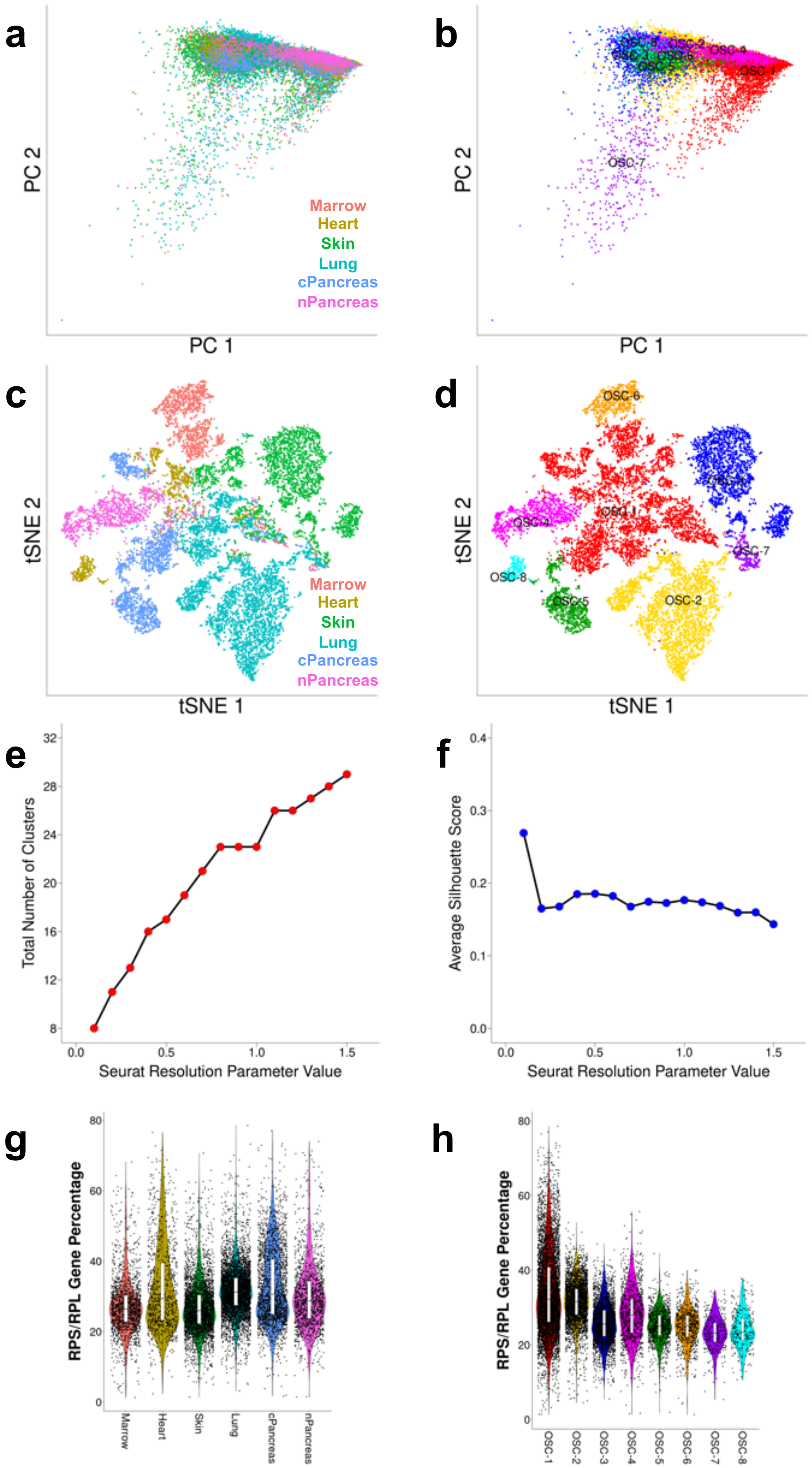
a
b
c
d
e
f
g
h
PC 2
PC 1
tSNE 2
tSNE 1
Marrow
Heart
Skin
Lung
cPancreas
nPancreas
OSC-1
OSC-2
OSC-4
OSC-5
OSC-6
OSC-7
OSC-8
Total Number of Clusters
Average Silhouette Score
Seurat Resolution Parameter Value
RPS/RPL Gene Percentage
OSC-3

Supp Fig 7 Dimensionality reduction and analysis of clustering results of re-normalized and re-clustered OSCs isolated from 3D *in vitro* microvessel network dataset. **a** PCA plot of OSC dataset separated by microvessel network type (Marrow, Heart, Skin, Lung, cPancreas, nPancreas). **b** PCA plot of OSC dataset separated by cluster identity (OSC-1, OSC-2, OSC-3, OSC-4, OSC-5, OSC-6, OSC-7, OSC-8). **c** tSNE plot of OSC dataset separated by microvessel network type. **d** tSNE plot of OSC dataset separated by cluster identity. **e** Scatterplot of resultant number of cell clusters to Seurat resolution parameter value (values between 0.1-1.5). **f** Scatterplot of average silhouette score for clusters as determined by Seurat resolution parameter (values between 0.1-1.5). **g** Violin plot of percentage of ribosomal genes (percentage of gene expression by *RPS* and *RPL* genes per cell) grouped by microvessel network type. **h** Violin plot of percentage of ribosomal genes (percentage of gene expression by *RPS* and *RPL* genes per cell) grouped by OSC cluster identity.

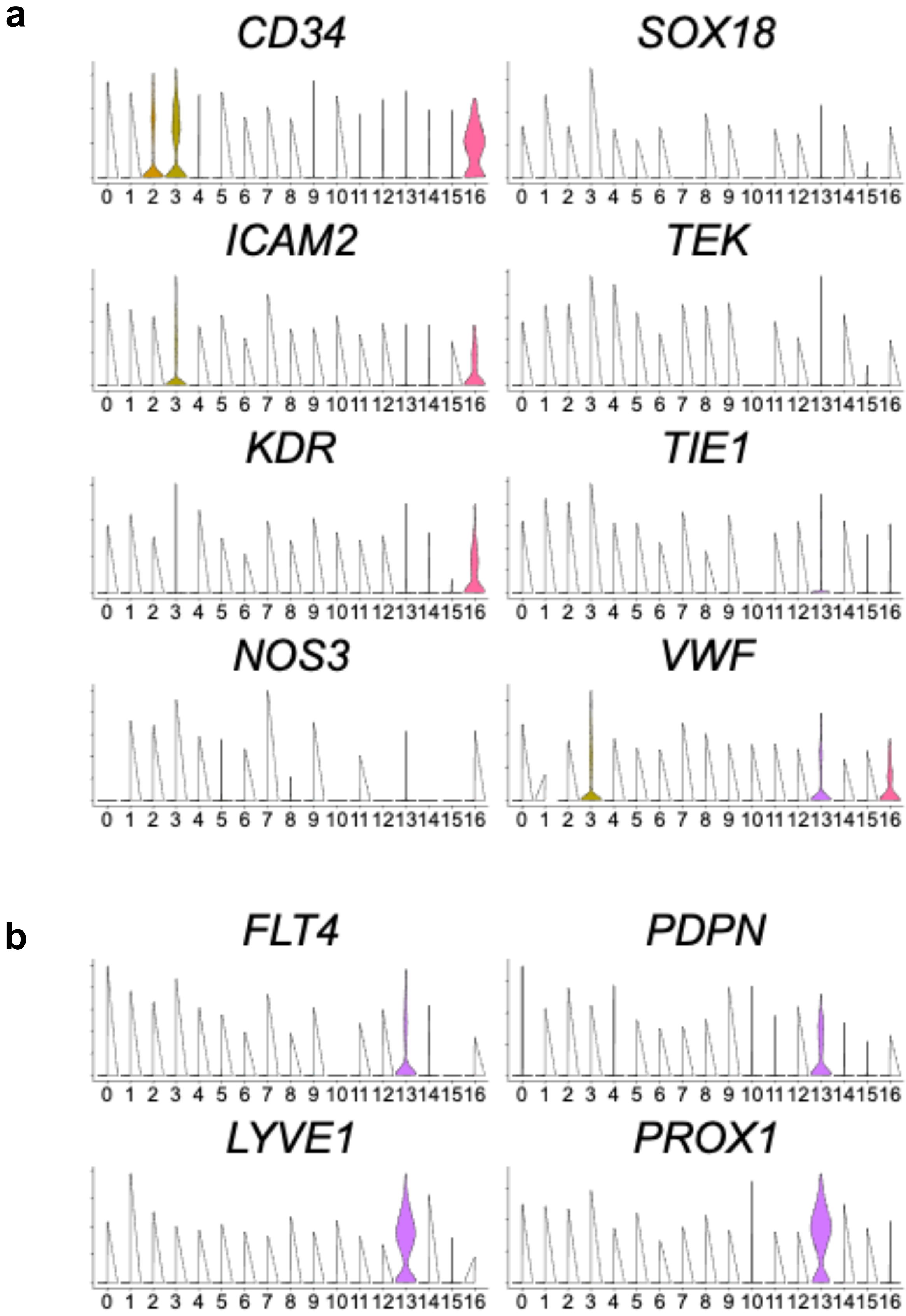
a
CD34
SOX18
ICAM2
TEK
KDR
TIE1
NOS3
VWF
b
FLT4
PDPN
LYVE1
PROX1
0 1 2 3 4 5 6 7 8 9 10 11 12 13 14 15 16

Supp Fig 8 Additional violin plots of EC-related genes from clusters identified in skin *in vivo* dataset (data from Solé-Boldo, *et al.*) identifies a population of lymphatic ECs to be excluded from downstream analysis [2]. **a** Violin plots of EC-related genes *CD34, ICAM2, KDR, NOS3, SOX18, TEK, TIE1,* and *VWF*. **B** Violin plots of lymphatic EC-related genes *FLT4*, *LYVE1, PDPN,* and *PROX1*.

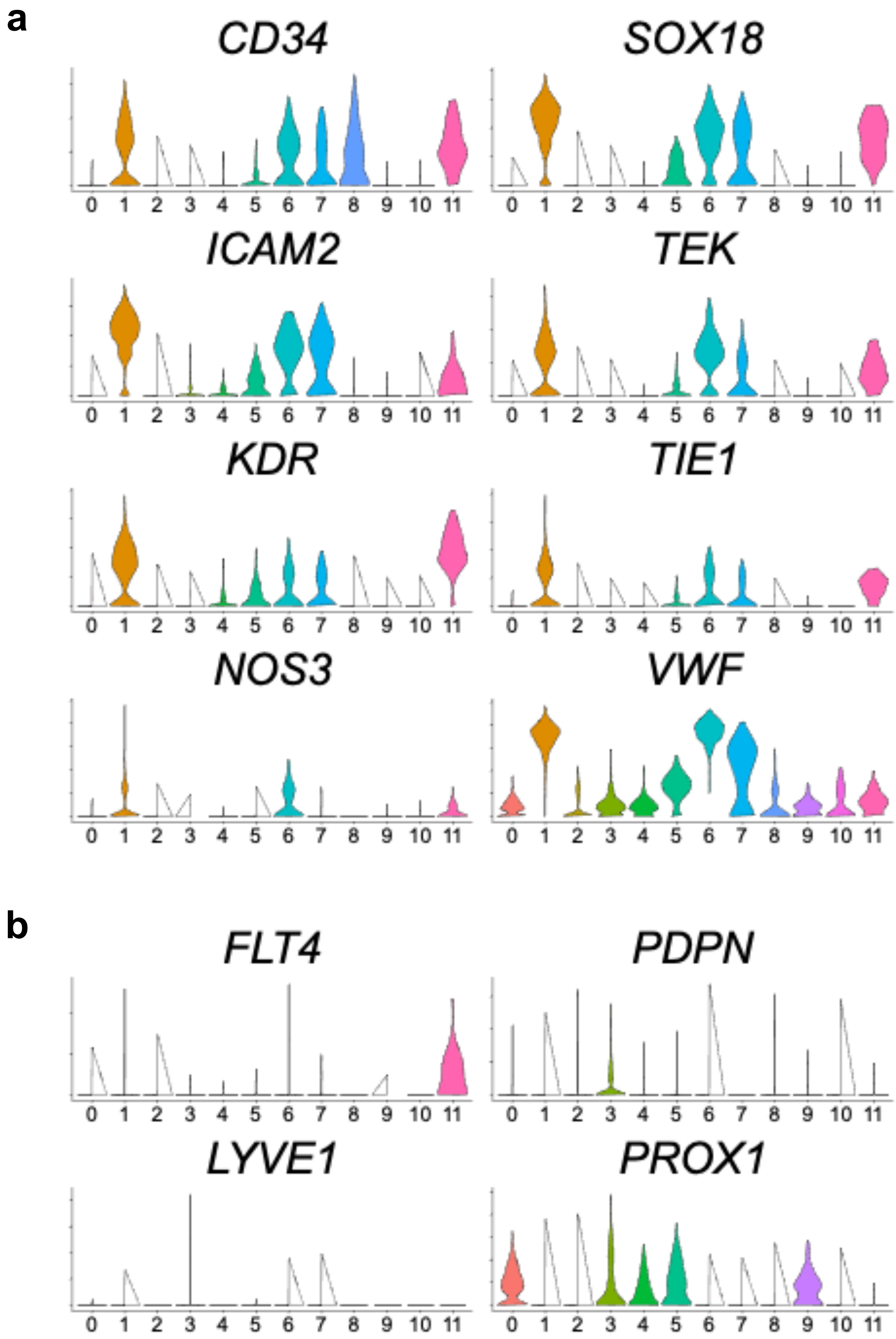
a
CD34
SOX18
ICAM2
TEK
KDR
TIE1
NOS3
VWF
b
FLT4
PDPN
LYVE1
PROX1
0 1 2 3 4 5 6 7 8 9 10 11

Supp Fig 9 Additional violin plots of EC-related genes from clusters identified in heart *in vivo* dataset (data from Tabula Sapiens, *et al.*) [1]. **a** Violin plots of EC-related genes *CD34, ICAM2, KDR, NOS3, SOX18, TEK, TIE1,* and *VWF*. **B** Violin plots of lymphatic EC-related genes *FLT4*, *LYVE1, PDPN,* and *PROX1*.

a

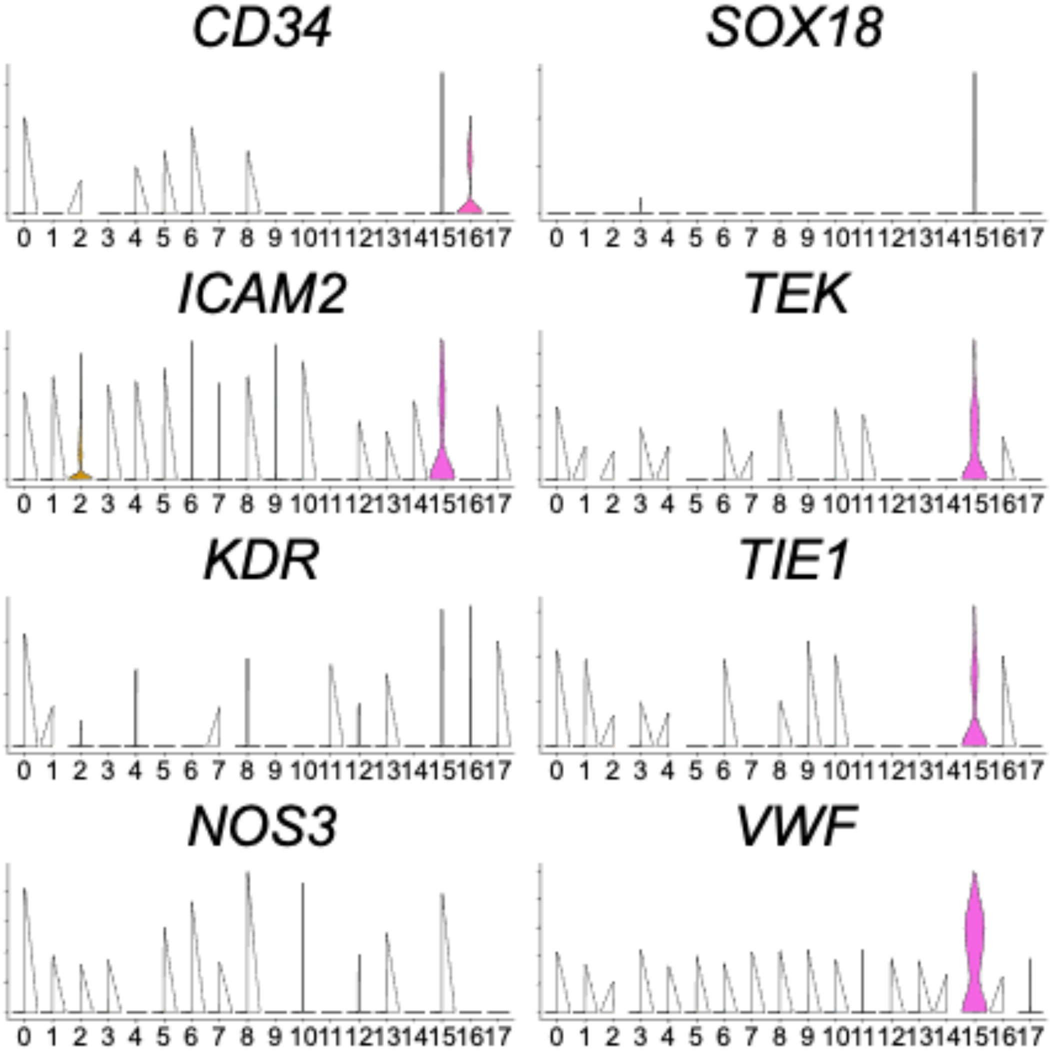

b

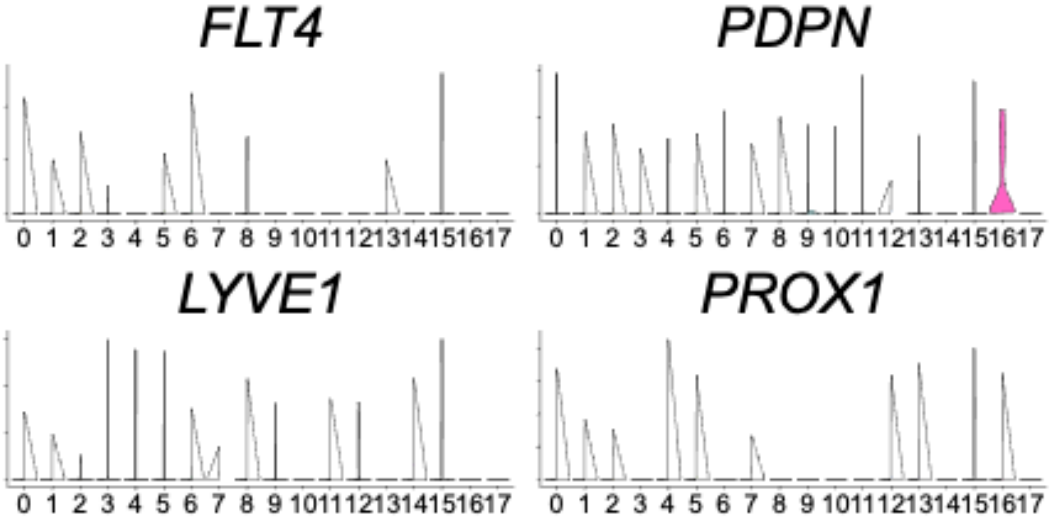

Supp Fig 10 Additional violin plots of EC-related genes from clusters identified in lung *in vivo* dataset (data from Schupp, *et al*) [3]. **a** Violin plots of EC-related genes *CD34, ICAM2, KDR, NOS3, SOX18, TEK, TIE1,* and *VWF*. **B** Violin plots of lymphatic EC-related genes *FLT4*, *LYVE1, PDPN,* and *PROX1*.

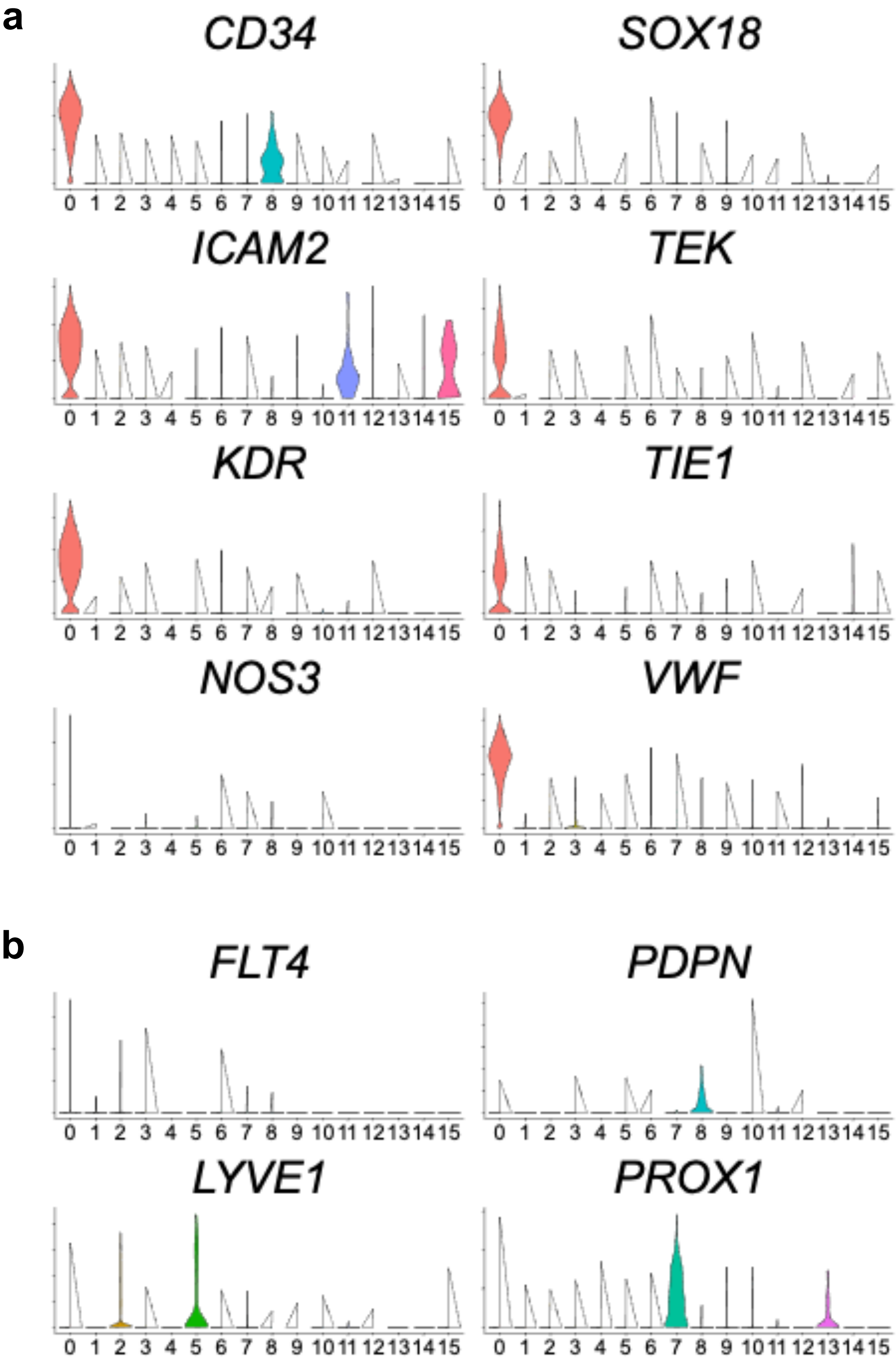
a
CD34
SOX18
ICAM2
TEK
KDR
TIE1
NOS3
VWF
b
FLT4
PDPN
LYVE1
PROX1
0 1 2 3 4 5 6 7 8 9 10 11 12 13 14 15

Supp Fig 11 Additional violin plots of EC-related genes from clusters identified in normal pancreas *in vivo* dataset (data from Tabula Sapiens, *et al*.) [1]. **a** Violin plots of EC-related genes *CD34, ICAM2, KDR, NOS3, SOX18, TEK, TIE1,* and *VWF*. **B** Violin plots of lymphatic EC-related genes *FLT4*, *LYVE1, PDPN,* and *PROX1*.

Vendor	Cat #	Fluorophore	Target	Vol. per 100 µL Test (µL)
BD Biosciences	560411	FITC	CD144/ VE-Cadherin	20
BioLegend	303116	APC	CD31/ PECAM1	5
BioLegend	328122	BV 421	CD90/ THY1	5
BioLegend	323606	PE	PDGFRβ/ CD140b	5
ThermoFisher	L10119	Fixable Near IR	Live/Dead	1

Supp Table 1 Antibodies used in the flow cytometry characterization of cell monolayers prior to 3D fibrin hydrogel culture from Supp. Fig. 1.

Vendor	Cat #	Species	Target	Fluorophore	Dilution
ThermoFisher	13031982	Mouse	CD31/ PECAM1	N/A	1:100
ThermoFisher	A21427	Rabbit	Anti-Mouse	Alexa Fluor 555	1:400

Supp Table 2 Antibodies used in the immunofluorescent characterization of microvessel networks from 3D *in vitro* fibrin hydrogels from Fig. 1.

Name	Environment	Version
Ubuntu	Linux	20.04.4
CellRanger	Linux	5.0.0
GRCh38	Linux	3.0.0
base	R	4.0.3
Matrix	R	1.4-1
org.HS.eg.db	R	3.12.0
topGO	R	2.42.0
SparseM	R	1.81
GO.db	R	3.12.1
circlize	R	0.4.14
AnnotationDbi	R	1.52.0
graph	R	1.68.0
stringr	R	1.4.0
SeuratWrappers	R	0.3.0
monocle3	R	1.0.0
SingleCellExperiment	R	1.12.0
SummarizedExperiment	R	1.20.0
GenomicRanges	R	1.42.0
GenomeInfoDb	R	1.26.7
IRanges	R	2.24.1
S4Vectors	R	0.28.1
MatrixGenerics	R	1.2.1
matrixStats	R	0.61.0
NMF	R	0.24.0
cluster	R	2.1.3
rngtools	R	1.5.2
pkgmaker	R	0.32.2
registry	R	0.5-1
CellChat	R	1.1.3
Biobase	R	2.50.0
BiocGenerics	R	0.36.1
igraph	R	1.3.0
cowplot	R	1.1.1
ggplot2	R	3.3.5
patchwork	R	1.1.1
dplyr	R	1.0.8
SeuratObject	R	4.0.4
Seurat	R	4.1.0
svglite	R	2.1.0
forcats	R	0.5.1

Supp Table 3 System version information for Linux command line and relevant packages from R computing environment.

#	EC-1	EC-2	EC-3	EC-4	EC-5
1	*MT2A*	*RGCC*	*NEAT1*	*HIST1H1B*	*MALAT1*
2	*MT1E*	*IL32*	*MT-CO2*	*HIST1H4C*	*NEAT1*
3	*TXN*	*LXN*	*HSPA5*	*UBE2C*	*VWF*
4	*SERPINE1*	*CDC42EP5*	*APLP2*	*HIST1H2AG*	*COL4A1*
5	*SH3BGRL3*	*SOX18*	*MT-CYB*	*HIST1H1C*	*HSPG2*
6	*RPL22L1*	*PGF*	*THBS1*	*HIST1H1D*	*COL4A2*
7	*BOLA3*	*TMSB4X*	*HSP90B1*	*CKS2*	*TCIM*
8	*HMGA1*	*TPT1*	*MT-ATP6*	*HMGB2*	*COL18A1*
9	*S100A6*	*RPS27*	*MALAT1*	*HIST1H3B*	*DEPP1*
10	*ANXA2*	*BTG1*	*CTGF*	*HIST1H1E*	*XIST*
11	*POLR2E*	*PNRC1*	*CYR61*	*HIST2H2AC*	*PECAM1*
12	*PRDX1*	*GNG11*	*AHNAK*	*CDK1*	*ARGLU1*
13	*GSTP1*	*RPL10*	*EDN1*	*CCNB1*	*CLDN5*
14	*PFN1*	*C12orf57*	*MT-CO3*	*TOP2A*	*WSB1*
15	*FKBP11*	*CALM1*	*HHIP*	*TPX2*	*TCF4*
16	*ADIRF*	*NOP53*	*MT-CO1*	*KPNA2*	*RHOB*
17	*LDHA*	*RPL28*	*ITGB1*	*NUSAP1*	*UACA*
18	*PSMD2*	*RPS28*	*PTX3*	*TUBB4B*	*PXDN*
19	*PPIA*	*RPL13*	*CRIM1*	*RRM2*	*MACF1*
20	*OAZ1*	*COMMD6*	*CLEC14A*	*TUBA1C*	*PNISR*
21	*MLLT11*	*RPL34*	*MT-ND4*	*CKS1B*	*SPTBN1*
22	*UROD*	*RPL26*	*ENG*	*AURKB*	*CLEC14A*
23	*ENO1*	*RPS4X*	*EFEMP1*	*UBE2S*	*LAMB1*
24	*FKBP3*	*RPS8*	*CKAP4*	*DLGAP5*	*THBS1*
25	*SRGN*	*RPS15A*	*MT-ND3*	*CDC20*	*NRP2*
26	*MYL12B*	*RPL39*	*SLC3A2*	*UBE2T*	*CDH5*
27	*S100A11*	*RPS26*	*SLC7A11*	*TUBA1B*	*UTRN*
28	*EFHD2*	*SELENOW*	*PRSS23*	*HIST1H4E*	*MYH9*
29	*VPS29*	*RPL15*	*CLDN11*	*SMC4*	*MMRN1*
30	*SDCBP*	*OST4*	*FLNB*	*H2AFZ*	*GABPB1-AS1*
31	*DKK1*	*RPS14*	*NR2F2*	*H2AFX*	*RSRP1*
32	*VEPH1*	*ZFAS1*	*MACF1*	*TYMS*	*HLA-B*
33	*TAF10*	*CCDC85B*	*AKAP12*	*PTTG1*	*KIAA1551*
34	*UCHL3*	*RPS3A*	*MMRN1*	*BIRC5*	*LUC7L3*
35	*NDUFA3*	*RPL37*	*APP*	*G0S2*	*RNF213*
36	*MYL6*	*RPL30*	*MCAM*	*PCLAF*	*PLK2*
37	*NDUFB6*	*GUK1*	*HMOX1*	*HMGN2*	*DLL4*
38	*IL1RL1*	*RPL32*	*ITGA5*	*STMN1*	*SOX4*
39	*NDUFAF2*	*RPL22*	*CD59*	*HMGB1*	*APP*
40	*CTNNAL1*	*RPS19*	*MT-ND2*	*RANBP1*	*RGCC*

Supp Table 4 Top 40 differentially expressed genes for each EC cluster, ordered by fold change over all other clusters (as determined by Seurat). Top 10 differentially genes for each cluster are shown in Fig. 4E.

#	OSC-1	OSC-2	OSC-3	OSC-4	OSC-5	OSC-6	*OSC-7*	OSC-8
1	*CCND1*	*CLU*	*SUSD2*	*TIMP1*	*AREG*	*IGFBP2*	*CENPF*	*COL3A1*
2	*ANP32B*	*MT1X*	*APOE*	*IGFBP3*	*CXCL1*	*PTX3*	*CKS2*	*COL1A1*
3	*DYNC1LI2*	*ADIRF*	*COL4A1*	*TGFBI*	*CXCL8*	*CFD*	*PTTG1*	*COL1A2*
4	*MAP4*	*DCN*	*S100A4*	*MT3*	*SERPINE1*	*FN1*	*UBE2S*	*IGFBP7*
5	*HNRNPA1*	*GLUL*	*NDUFA4L2*	*LOX*	*ANGPTL4*	*PLAT*	*HMGB2*	*C7*
6	*MAP1B*	*CD9*	*CRYAB*	*COL1A1*	*PAPPA*	*COL6A3*	*TOP2A*	*MGP*
7	*S100A11*	*ADH1B*	*COL4A2*	*CTGF*	*GDF15*	*DUSP23*	*CCNB1*	*COL5A2*
8	*SH3BGRL3*	*MT1E*	*BGN*	*GLRX*	*HSPA5*	*RPS26*	*ASPM*	*FBN1*
9	*NPM1*	*SFRP1*	*CCL2*	*CTHRC1*	*RALA*	*COL8A1*	*UBE2C*	*PAPPA*
10	*PTPN11*	*CAMK2N1*	*TAGLN*	*LMCD1*	*GREM1*	*STC2*	*HIST1H4C*	*COL6A3*
11	*ZEB1*	*ID1*	*ID3*	*F3*	*SOD2*	*CCDC68*	*TPX2*	*CXCL2*
12	*UACA*	*ADAMTS1*	*COL6A3*	*FBXO32*	*INHBA*	*GAS6*	*H2AFZ*	*COL6A1*
13	*SET*	*DUSP1*	*COL6A1*	*COL3A1*	*HMGA2*	*LEPR*	*CKAP2*	*NDUFA4L2*
14	*MAP4K4*	*PHLDA1*	*NR4A1*	*CCDC80*	*SLC16A3*	*SRGN*	*STMN1*	*COL5A1*
15	*KIF1C*	*C1S*	*XIST*	*SPARC*	*PDLIM4*	*TXNIP*	*CDKN3*	*CXCL1*
16	*PALLD*	*MALAT1*	*LY6K*	*PLOD2*	*P4HA2*	*PLA2G16*	*TUBB4B*	*SPARC*
17	*TCEAL4*	*C1R*	*CYR61*	*SERPINE1*	*MME*	*CHST2*	*MKI67*	*COL6A2*
18	*CFL1*	*CCDC85B*	*COL6A2*	*IER3*	*CDKN1A*	*ITGBL1*	*SMC4*	*LOXL2*
19	*FKBP1A*	*PLIN2*	*C11orf96*	*COL1A2*	*CXCL2*	*PIP*	*NUSAP1*	*COL11A1*
20	*C12orf75*	*CEBPD*	*HLA-DRB1*	*COL5A1*	*HILPDA*	*DSEL*	*CENPE*	*CXCL3*
21	*MYL9*	*TBX3*	*TNC*	*NNMT*	*PLOD2*	*STMN2*	*CKS1B*	*CTGF*
22	*PFN1*	*MT1G*	*ACTA2*	*GREM1*	*SPON2*	*WNT5B*	*ARL6IP1*	*HTRA1*
23	*PPIA*	*NFKBIA*	*LOXL2*	*CTSC*	*VCAN*	*CCPG1*	*HMMR*	*PCOLCE*
24	*HSP90AA1*	*SELENOP*	*JUNB*	*THBS1*	*G0S2*	*CPM*	*BIRC5*	*COL4A1*
25	*MYL12A*	*IGFBP6*	*EMP1*	*POSTN*	*UCHL1*	*THBS1*	*TUBA1C*	*C11orf96*
26	*OAZ1*	*SPON2*	*COL8A1*	*HSD17B1*	*NDRG1*	*NPR3*	*KPNA2*	*CRLF1*
27	*MYL12B*	*MT1M*	*EPS8*	*CCL20*	*UGCG*	*TFPI*	*KIF20B*	*SRGN*
28	*TFPI2*	*TXNIP*	*PDGFRB*	*HLA-B*	*PLAUR*	*LEPROT*	*TUBA1B*	*EFEMP1*
29	*YBX1*	*SRPX*	*FOS*	*INHBA*	*ERO1A*	*NUPR1*	*CCNB2*	*MT-CO1*
30	*PTMS*	*GPX3*	*MT1M*	*TPM1*	*MEG3*	*RTN4*	*CDK1*	*GSTT2B*
31	*NCL*	*CTSK*	*SCUBE3*	*TFPI2*	*CCL20*	*DKK1*	*CDC20*	*ID3*
32	*TMSB4X*	*LGALS3*	*SERPINF1*	*HSD11B1*	*METRNL*	*GALNT15*	*ANLN*	*COL5A3*
33	*ATP5F1E*	*PDE5A*	*NID1*	*FKBP11*	*DCBLD2*	*TRIB3*	*DLGAP5*	*SERPINH1*
34	*CYCS*	*TBX2-AS1*	*NOTCH3*	*SAT1*	*TNFAIP6*	*DNAJC15*	*AURKA*	*POSTN*
35	*TPM4*	*NR2F1*	*TINAGL1*	*RGCC*	*CALR*	*EPSTI1*	*HMGB1*	*ACTA2*
36	*EIF5B*	*CKB*	*NR2F2*	*HIF1A*	*FAM162A*	*NETO2*	*MAD2L1*	*MT-CYB*
37	*RPL22L1*	*F2R*	*MALAT1*	*LDHA*	*VEGFA*	*HTRA1*	*BUB3*	*HGF*
38	*HNRNPA3*	*CXCL3*	*LAMC1*	*RECK*	*CXCL6*	*GADD45A*	*SGO2*	*COL7A1*
39	*TPM2*	*NEAT1*	*ID1*	*KDELR3*	*HIF1A*	*SLC3A2*	*JPT1*	*CXCL8*
40	*TPM3*	*PTN*	*HTRA1*	*KDELR2*	*KRT18*	*LMO7*	*CENPF*	*THBS2*

Supp Table 5 Top 40 differentially expressed genes for each OSC cluster, ordered by fold change over all other clusters (as determined by Seurat).

#	ECFC Monolayer	Skin 3D *in vitro* microvessel network ECs	Skin *in vivo* ECs
1	*MT2A*	*PHGDH*	*CD74*
2	*LGALS1*	*TAGLN*	*ZFP36*
3	*VIM*	*DDX10*	*HLA-DRA*
4	*RPLP1*	*MPZL2*	*GNB2L1*
5	*TMSB10*	*COL1A1*	*DARC*
6	*EEF1A1*		*SPARCL1*
7	*FTL*		*SOD2*
8	*S100A6*		*KRT14*
9	*GAPDH*		*JUNB*
10	*RPS2*		*SOCS3*
11	*RPL10*		*HLA-DRB1*
12	*RPS12*		*AQP1*
13	*RPS18*		*FOS*
14	*RPL13*		*NFKBIA*
15	*RPL41*		*S100A4*
16	*RPS3*		*SPRY1*
17	*FTH1*		*SELE*
18	*RPS19*		*RGS16*
19	*MT-CO2*		*IL6*
20	*MALAT1*		*CXCL2*

Supp Table 6 Top 10 differentially expressed genes for each culture condition from the Skin EC comparisons (Fig. 6A), ordered by fold change over all other clusters (as determined by Seurat). Only 5 genes were determined to be differentially expressed between the Skin 3D *in vitro* microvessel network ECs compared to all other cells.

#	ECFC Monolayer	Heart 3D *in vitro* microvessel network ECs	Heart *in vivo* ECs
1	*THBS1*	*RPS2*	*MT-RNR2*
2	*APLN*	*RPLP0*	*SPARCL1*
3	*HHIP*	*RPL13A*	*FABP4*
4	*MCAM*	*RPS6*	*TIMP3*
5	*CYR61*	*RPL3*	*MT-RNR1*
6	*KRT7*	*RPL12*	*TXNIP*
7	*CTGF*	*RPL15*	*H19*
8	*CD99*	*RPL6*	*VWF*
9	*SERPINE1*	*RPL23A*	*CD36*
10	*PLAT*	*RPL5*	*CD74*
11	*PTX3*	*RPS13*	*MYL7*
12	*PPP1R14B*	*RPL27A*	*F8*
13	*TUBA1C*	*RPL7*	*ID1*
14	*AKAP12*	*RPL31*	*MTATP6P1*
15	*RALA*	*SLC25A6*	*A2M*
16	*HMGA1*	*COL1A1*	*ACTC1*
17	*MT2A*	*RPL22L1*	*STOM*
18	*ANKRD1*	*TKT*	*FLT1*
19	*CCND1*	*NNMT*	*FCN3*
20	*EDN1*	*RPL17*	*AQP1*

Supp Table 7 Top 10 differentially expressed genes for each culture condition from the Heart EC comparisons (Fig. 6B), ordered by fold change over all other clusters (as determined by Seurat).

#	ECFC Monolayer	Lung 3D *in vitro* microvessel network ECs	Lung *in vivo* ECs
1	*AKAP12*	*FTL*	*TIMP3*
2	*KRT7*	*GAPDH*	*GALNT18*
3	*TUBA1A*	*RPLP0*	*AC073861.1*
4	*APLN*	*RPL12*	*SPARCL1*
5	*HHIP*	*FTH1*	*PTPRM*
6	*THBS1*	*TPT1*	*KIAA1217*
7	*TUBA1B*	*RPL17*	*AQP1*
8	*JPT1*	*DCN*	*PRKG1*
9	*NES*	*TIMP1*	*AFF3*
10	*ANPEP*	*TXN*	*RALGAPA2*
11	*TUBB4B*	*IFITM2*	*MT-RNR2*
12	*MT-CO2*	*TKT*	*SLCO2A1*
13	*MT2A*	*GLRX*	*PTPRG*
14	*MCAM*	*RAB13*	*LDB2*
15	*MT-ND3*	*RPS4Y1*	*ADGRL2*
16	*ANKRD1*	*MIF*	*CCL21*
17	*TUBA1C*	*NQO1*	*FCN3*
18	*PLAT*	*PTGR1*	*HSPA1A*
19	*TFPI2*	*BNIP3*	*EPAS1*
20	*HIST1H4C*	*HMOX1*	*MGP*

Supp Table 8 Top 10 differentially expressed genes for each culture condition from the Lung EC comparisons (Fig. 6C), ordered by fold change over all other clusters (as determined by Seurat).

#	ECFC Monolayer	nPancreas 3D *in vitro* microvessel network ECs	nPancreas *in vivo* ECs
1	*AKAP12*	*RPL12*	*SPARCL1*
2	*APLN*	*RPS2*	*MT-RNR2*
3	*MCAM*	*RPLP0*	*TXNIP*
4	*HHIP*	*RPS8*	*MT-RNR1*
5	*MAP1B*	*GAPDH*	*CLPS*
6	*CCND1*	*RPS19*	*PLVAP*
7	*ANKRD1*	*ACTG1*	*CD74*
8	*CD99*	*RPS13*	*ID1*
9	*CTGF*	*RPL31*	*PRSS1*
10	*THBS1*	*RPS16*	*HLA-B*
11	*PTX3*	*SLC25A6*	*FABP5*
12	*HMMGA1*	*COL1A1*	*PRSS2*
13	*KRT18*	*COL1A2*	*A2M*
14	*LGALS1*	*SERPINE1*	*TIMP3*
15	*TUBA1A*	*TPM2*	*REG1A*
16	*PPP1R14B*	*TIMP1*	*ITM2B*
17	*TUBA1C*	*EIF4EBP1*	*CD36*
18	*RALA*	*EFEMP1*	*GSN*
19	*TUBA1B*	*FN1*	*HLA-C*
20	*APLP2*	*ZFAS1*	*TGFBR2*

Supp Table 9 Top 10 differentially expressed genes for each culture condition from the nPancreas EC comparisons (Fig. 6B), ordered by fold change over all other clusters (as determined by Seurat).

References

1. THE TABULA SAPIENS CONSORTIUM (2022) The Tabula Sapiens: A multiple-organ, single-cell transcriptomic atlas of humans. Science 376:eabl4896. https://doi.org/10.1126/science.abl4896

2. Solé-Boldo L, Raddatz G, Schütz S, et al (2020) Single-cell transcriptomes of the human skin reveal age-related loss of fibroblast priming. Communications Biology 2020 3:1 3:1–12. https://doi.org/10.1038/s42003-020-0922-4

3. Schupp JC, Adams TS, Jr CC, et al (2021) Integrated Single-Cell Atlas of Endothelial Cells of the Human Lung. Circulation 144:286–302. https://doi.org/10.1161/CIRCULATIONAHA.120.052318
